# An efficient *Agrobacterium tumefaciens*-mediated transformation method for *Simplicillium subtropicum* (Hypocreales: Cordycipitaceae)

**DOI:** 10.1590/1678-4685-GMB-2021-0073

**Published:** 2021-10-01

**Authors:** Nicolau Sbaraini, Mariana Vieira Tomazett, Augusto Bartz Penteriche, Relber Aguiar Gonçales, Matheus da Silva Camargo, Alexandre Melo Bailão, Clayton Luiz Borges, Augusto Schrank, Célia Maria de Almeida Soares, Charley Christian Staats

**Affiliations:** 1 Universidade Federal do Rio Grande do Sul, Centro de Biotecnologia, Programa de Pós-graduação em Biologia Celular e Molecular, Porto Alegre, RS, Brazil.; 2 Rede Avançada em Biologia Computacional (RABICÓ), Petrópolis, RJ, Brazil.; 3 Universidade Federal de Goiás, Instituto de Ciências Biológicas, Laboratório de Biologia Molecular, Goiânia, GO, Brazil.; 4 University of Minho, School of Medicine, Life and Health Sciences Research Institute (ICVS), Braga, Portugal.; 5 ICVS/3B’s - PT Government Associate Laboratory, Braga/Guimarães, Portugal.

**Keywords:** Simplicillium, Simplicillium subtropicum, Agrobacterium tumefaciens, Katushka, *Agrobacterium tumefaciens*-mediated transformation

## Abstract

Filamentous fungi are the organisms of choice for most industrial biotechnology. Some species can produce a variety of secondary metabolites and enzymes of commercial interest, and the production of valuable molecules has been enhanced through different molecular tools. Methods for genetic manipulation and transformation have been essential for the optimization of these organisms. The genus *Simplicillium* has attracted increased attention given several potential biotechnological applications. The *Simplicillium* genus harbors several entomopathogenic species and some isolates have been explored for bioremediation of heavy metal contaminants. Furthermore, the myriad of secondary metabolites isolated from *Simplicillium* spp. render these organisms as ideal targets for deep exploration and further biotechnological mining possibilities. However, the lack of molecular tools hampered the exploration of this genus. Thus, an *Agrobacterium tumefaciens*-mediated transformation method was established for *Simplicillium subtropicum*, employing the far-red fluorescent protein TURBOFP635/Katushka, as a visual marker, and the selection marker *SUR* gene, that confers resistance to chlorimuron ethyl. Notably, one round of transformation using the established method yielded almost 400 chlorimuron resistant isolates. Furthermore, these transformants displayed mitotic stability for, at least, five generations. We anticipate that this method can be useful for deep molecular exploration and improvement of strains in the *Simplicillium* genus.

## Introduction

The family Cordycipitaceae harbors filamentous fungi of commercial and scientific interest. The most recognizable member of this family, the *Beauveria* genus, embraces entomopathogenic species (Zimmermann, 2007). Several commercial formulations of *Beauveria bassiana* are available worldwide for agricultural pests biological control (Faria and Wraight, 2007). Similarly, species from the *Cordyceps* genus are retailed, given several potential health benefits (Tuli *et al*., 2014). Furthermore, some species from this family can be mycoparasitic (Kepler *et al*., 2017). For instance, the dry bubble disease caused by *Lecanicillium fungicola* is a persistent problem in the cultivation of *Agaricus bisporus* (Berendsen *et al*., 2010), while *Akanthomyces psalliotae* is an entomopathogenic, mycoparasitic, and nematophagous fungus (Harm *et al*., 2018).

The earliest diverging lineage in the family Cordycipitaceae is the *Simplicillium* genus (Kepler *et al*., 2017). This genus has attracted interest for several potential applications, ranging from biological control to remediation by toxic metal accumulation. Some mycoparasitic *Simplicillium* species can be employed for biological control of oomycetes and fungi, as *Simplicillium lamellicola* and *Simplicillium lanosoniveum*, while the latter species can also be a plant pathogen (Chen *et al*., 2008; Ward *et al*., 2012; Shin *et al*., 2017). *Simplicillium chinense* has been explored for biosorption of cadmium and lead (Jin *et al*., 2019; Jin *et al*., 2020). Additionally, this species can ameliorate the phytoremediation performance of the water-reed *Phragmites communis* (Jin *et al*., 2019).

Several secondary metabolites have been isolated from *Simplicillium* spp. Simplicilliumtides, from *Simplicillium obclavatum*, possess a broad range of biological activities, including antibacterial, antifungal, antiviral, antifouling, cytotoxic, as well as acetylcholinesterase inhibitory activity (Youssef *et al*., 2019). Aogacillins A and B, produced by *Simplicillium* sp. FKI-5985, circumvent arbekacin resistance in methicillin-resistant *Staphylococcus aureus* (Takata *et al*., 2013). Verlamelins A and B and sinulariapeptide A, isolated from a soft coral-associated *Simplicillium* sp., showed antifungal activity against *Pyricularia oryzae* and *Colletotrichum asianum*, respectively (Dai *et al*., 2018). Simpotentin, isolated from the culture broth of *Simplicillium minatense*, has been described as a potentiator of amphotericin B activity against *Candida albicans* and *Cryptococcus neoformans* (Uchida *et al*., 2019). Furthermore, several compounds isolated from *Simplicillium lanosoniveum* have shown antibacterial, antifungal and phosphodiesterase 5 inhibitory activity (Rukachaisirikul *et al*., 2019).

Contrasting with the biotechnological potential of the *Simplicillium* genus, there is no method for genetic modification of these species. Thus, to address this obstacle, a highly efficient *Agrobacterium tumefaciens*-mediated transformation (ATMT) method was standardized for *Simplicillium subtropicum*, employing, as an *in vivo* tag, the reporter far-red fluorescent protein TURBOFP635/ Katushka (Kat) (Shcherbo *et al*., 2007), and, as a selection marker, the *SUR* gene.

## Material and Methods

### Strains and culture media

*S. subtropicum* strain IBCB 79 was originally isolated from a dead *Leptopharsa heveae* collected in Mato Grosso, Brazil. This strain is deposited in the fungal collection “Coleção de Culturas de Entomopatógenos Oldemar Cardim Abreu” located in São Paulo, Brazil. Before subsequent experiments, this strain was grown at 28 °C in solid Cove’s Complete Medium (MCc), as previously described (Sbaraini *et al*., 2019). Maintenance of transformants were in solid Cove’s Medium (MC), as described by Sbaraini and coworkers (2019), and 10 μg/mL of Chlorimuron Ethyl (CE) was employed. *Escherichia coli* TG2 was employed in routine cloning, and *Agrobacterium tumefaciens* strains EHA105 and LBA1100 were employed to perform the ATMT of *S. subtropicum* strain IBCB 79. Bacteria were obtained from the laboratory’s collection and maintained in Luria-Bertani (LB) medium with the appropriate antibiotics (Green and Sambrook, 2012).

### Species identification and phylogeny

To ensure that strain IBCB 79 is a member of the *Simplicillium* genus, the internal transcribed spacer (ITS) region was sequenced and analyzed. DNA was extracted employing the standard phenol/chloroform method (Green and Sambrook, 2012). PCR reaction was performed following the standard protocols. DNA sequencing was performed by ACTGene Análises Moleculares (Brazil, RS) employing Sanger sequencing (Applied Biosystems, AB3500). The sequence (MT822178) was amended together with other sequences employed in the proposed *Simplicillium* genus tree described previously (Crous *et al*., 2018). The DNA barcode sequences were subjected to alignment reliability analyses using GUIDANCE 2.0 (alignment is shown in Supplementary Data S1), using the PRANK algorithm for sequence alignment with 100 bootstrap replicates and variable gap penalties (Loytynoja and Goldman, 2010; Sela *et al*., 2015). Additionally, a GUIDANCE 2.0 score cutoff of 0.93 for site removal was employed (Sela *et al*., 2015). The phylogenetic reconstruction was conducted with PhyML 3.1 (Maximum Likelihood) with aLRT SH-like (approximate likelihood ratio test Shimodaira-Hasegawa) branch support estimation (Guindon *et al*., 2010), employing GTR+I+G as the evolutionary model.

### *Katushka* reporter plasmid construction

For expression of the *KAT* gene, first, the *Magnaporthe grisea* acetolactate synthase enconding gene (*SUR*), which confers resistance to CE (Lin *et al*., 2011), was PCR-amplified with primers pPZP_EcoRV_SUR_F and pPZP_EcoRV_SUR_R (Table S1) and introduced in the *Eco*RV site of the binary vector pPZP201BK (Walton *et al*., 2005), to generate the plasmid pPZP201BK::SUR. The isolation of the *SUR* gene (sulfonylurea resistance allele *M. grisea ILV*1) has been described previously (Sweigard *et al*., 1997). The *SUR* gene, along with its native promoter and terminator, was obtained from plasmid pCB1532 (kindly provided by Aline S. Romão-Dumaresq and Nicholas Talbot). The *Kat* coding sequence was PCR amplified with primers gpdA_CDSKat_F and TtrpC_CDSKat_Rfrom the plasmid pJAF15::H3P::Kat::H3T (i. e., the gene is under control of the Histone 3 promoter and terminator of *Cryptococcus neoformans*; the plasmid was kindly provided by Marilene Henning Vainstein) and cloned in the NcoI and *Bam*HI sites of the plasmid pAN::gpdA::BAR::TrpC, to generate the plasmid pAN::gpdA::Kat::TrpC (i. e., NcoI and *Bam*HI digestion released the *BAR* gene) (Haleva *et al*., 2020). The *Kat* gene expression cassette, gpdA::Kat::TrpC was PCR amplified with primers pPZP_HindIII_gpdA_Kat_F and pPZP_HindIII_trpC_Kat_R, and cloned in the *Eco*RV site of the plasmid pPZP201BK::SUR to generate the plasmid pPZP201BK::SUR::gpdA::Kat::TrpC (primers, plasmid map, and plasmid sequence were included as Table S1, Figure S1, and Supplementary Data S2, respectively). All cloning steps were performed employing the Hot fusion protocol (Fu *et al*., 2015).

### *Agrobacterium tumefaciens*-mediated transformation

The protocol used for the transformation of *S. subtropicum* was similar to the ATMT method used for *Aspergillus awamori* (Michielse *et al*., 2008), and *Paracoccidioides brasiliensis* (Almeida *et al*., 2007; Menino *et al*., 2012). Furthermore, these employed protocols were based on the work of Bundock *et al*. (1995), and de Groot *et al*. (1998). In brief, cultures of *A. tumefaciens* EHA105 or LBA1100 (carrying the *Katushka* plasmid), which were overnight grown in LB broth supplemented with antibiotics, were inoculated in 10 mL of freshly prepared induction medium (IM) supplemented with 400 μM acetosyringone (AS) and antibiotics. The cells were grown in IM at 28 °C and 180 rpm until reaching OD_600_ nm of 0.8-0.9. Concomitantly, five days old MCc plates presenting *S. subtropicum* growth were used to prepare a fresh spore suspension. The plates were washed with Tween 80 0.01 % (w/v) solution, the spores recovered and washed two times with liquid IM without antibiotics or AS. Spore counting was performed employing a hemocytometer and spore concentration was adjusted to the desired concentration (1 x 10^6^, 1 x 10^7^, or 1 x 10^8^ spores/mL) with liquid IM without antibiotics or AS. Subsequently, one hundred μL of the *A. tumefaciens* resulting growth was mixed with 50 μL of the *S. subtropicum* spore suspension. The *A.tumefaciens*-*S. subtropicum* mixture was kindly homogenized and pipetted over 0.45 μm Hybond N+ filter membranes disposed over solid IM supplemented with 400 μM AS and antibiotics. The mixture was left evaporating for 30 min in the dark before co-cultivation at 24 °C for 24, 48, and 72 h. After co-cultivation, the resulting growth (over the filter membranes), was scrapped in liquid MC supplemented with 200 µg/mL of cefotaxime and 10 µg/mL of CE and spread over MC plates supplemented with 200 µg/mL of cefotaxime and 10 µg/mL of CE. The plates were incubated at 28 °C until the transformants emergence. Potential transformants usually start to appear after 2 days. All transformations were performed in triplicates in two independent experiments. Emerging transformants were transferred to a new MC plate supplemented with 10 µg/mL CE for additional selection. Subsequently, selected transformants were cultured in MCc medium without the selection agent for five generations to evaluate mitotic stability.

### Screening of the transformants by fluorescent imaging

The first screening for Kat expression was carried out by qualitative detection of the far-red fluorescence using the Living Image 3.1 software in IVIS Lumina II (PerkinElmer). The parameters were set to 60 second exposure time, excitation at 535 nm and 465 nm (for background removal), using the dsRed emission filter. Not only transformant fungal colonies were inspected, as we also checked *A. tumefaciens* EHA105 harboring the plasmid pPZP201BK::SUR::gpdA::Kat::TrpC (i.e., the observed fluorescence could come from a bacterial contaminant rather than the selected mutants). Furthermore, intracellular expression of Kat was evaluated using the FLoid Cell Imaging Station (Thermo Fisher Scientific) with the red filter parameters (excitation: 586 nm; emission: 646 nm). Moreover, for detailed microscopic visualization of the Kat-fluorescence, the FLoid Cell Imaging Station was also employed. In this assay, wild-type and mutant strains were microcultured in MCc (28 °C, 4 days) and posteriorly inspected. 

Genomic DNA of potential transformants was extracted employing the phenol: chloroform method (Green and Sambrook, 2012). PCR to amplify *SUR* and *KAT* were also performed to evaluate the mutants (Primer sequences are described in Table S1). Subsequently, three selected mutants and the wild-type strain were evaluated by Southern blotting (Alkphos Direct Labeling and Detection System, GE Healthcare), to confirm the insertion of the *Kat* expression cassette and potentially evaluate the number of insertions in the genome. Genomic DNA was digested with StuI and further hybridized with a *KAT* CDS probe.

### Statistics

Statistical analyses were conducted with GraphPad Prism 6 (GraphPad software). Transformation efficiencies were plotted in number of isolated transformants. Effective transformation regimes were determined by one-way ANOVA with posthoc Tukey’s test (*p* < 0.01). The letters above bars indicate the statistical difference between transformation regimes.

## Results

### *S. subtropicum* phylogeny

**Figure 1 -:**
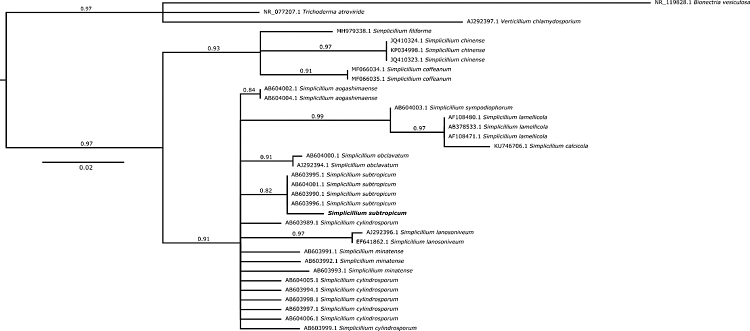
*Simplicillium subtropicum* species phylogenetic tree. A species tree based on ITS sequences was constructed to establish the relationships between the *Simplicillium* species with available ITS sequences and strain IBCB 79. *Bionectria vesiculosa*, *Trichoderma atroviride*, and *Verticillium chlamydosporium* entries were employed as outgroup and the tree was rooted in these species. Phylogenetic analysis was performed using Maximum-likelihood. Branch support values (aLRT SH-like supports) are associated with nodes. Polytomies were included when branch support values were lower than 0.80.

To characterize the genetic identity of strain IBCB 79, ITS sequencing and phylogeny were employed. This step was performed due to the inherent difficulty to distinguish *Simplicillium* isolates from *Sporothrix insectorum* isolates. The ITS sequence was compared with other *Simplicillium* sequences used to describe *Simplicillium filiforme*, a new endophytic species, recently described (Crous *et al*., 2018). The inferred phylogenetic tree supports the inclusion of the strain IBCB 79 as *S. subtropicum* (Figure 1). Originally isolated from soil in Japan (Nonaka *et al*., 2013), *S. subtropicum* has been explored for bioremediation of copper polluted areas, displaying the best results among few selected species (Ong *et al*., 2017).

### *Agrobacterium tumefaciens*-mediated transformation of *S. subtropicum*

**Figure 2 -:**
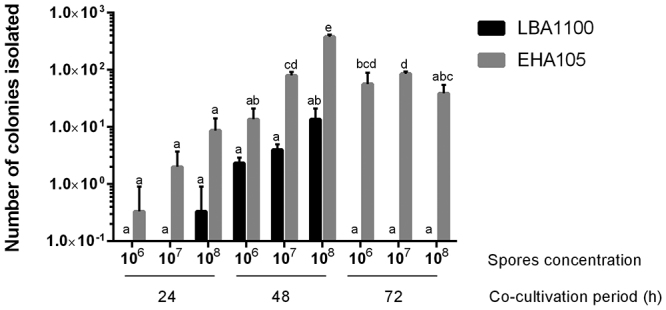
Transformation efficiency. To determine the most suitable transformation protocol for *S. subtropicum*, different strains of *A. tumefaciens* were evaluated (EHA105 and LBA1100), as well as different spore concentrations (1 x 10^6^, 1 x 10^7^, or 1 x 10^8^ spores/mL) and co-cultivation times (24, 48, and 72 h). The different letters above bars indicate statistical differences between transformation regimes according to one-way ANOVA analysis followed by posthoc Tukey’s test (*p* < 0.01).

For several filamentous fungi (e.g., *A. awamori* and *Paracoccidioides* spp.) the strain LBA1100 of *A. tumefaciens* is the most used for ATMT (Michielse *et al*., 2008; Fernandes *et al*., 2017), while, for species from Hypocreales order, the strains EHA105 or AGL1 are usually employed (Staats *et al*., 2007; Padilla-Guerrero and Bidochka, 2017). Thus, the *A. tumefaciens* strains EHA105 and LBA1100 were evaluated for *S. subtropicum* transformation. Furthermore, the effect of different spore concentrations, during co-cultivation, was also assessed. Finally, different co-cultivation periods (24, 48, and 72 h) were also evaluated. The best results were obtained employing *A. tumefaciens* strain EHA105 and 1 x 10^8^ spores/mL of *S. subtropicum* during 48 h of co-cultivation (Figure 2). Almost 400 transformants were obtained employing 1 x 10^8^ spores/mL, although, it is important to notice, that 1 x 10^7^ spores/mL can be more suitable for routine experiments since fewer spores are needed and transformants are easily isolated. A similar number of transformants were obtained employing 1 x 10^7^ spores/mL of *S. subtropicum* with 48 and 72 h of co-cultivation (Figure 2) Furthermore, the results discourage the use of *A. tumefaciens* strain LBA1100 for the transformation of *S. subtropicum*. Notably, transformants were not obtained when *A. tumefaciens* strain LBA1100 was co-cultured with *S. subtropicum* for 72 h (Figure 2).

### Kat-fluorescence detection and mitotic stability analysis

**Figure 3 -:**
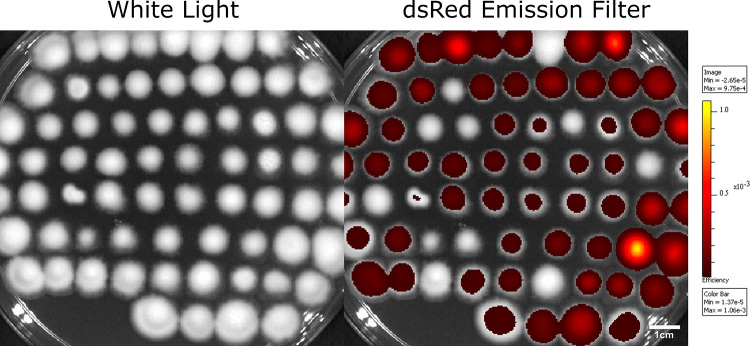
Macroscopic evaluation of far-red Kat-fluorescence. Fifty-eight of sixty-nine mutants evaluated displayed the far-red Kat-fluorescence (84 %). The parameters on Living Image 3.1 (implemented in the IVIS Lumina II equipment) were set to 60 second exposure time, excitation at 535 nm and 465 nm (for background removal), using the dsRed emission filter. Left Panel, White Light. Right Panel, dsRed emission filter.

Transformants from the ATMT experiments, selected based on CE resistance, were first evaluated for far-red Kat-fluorescence using the Living Image 3.1 software implemented in the IVIS Lumina II equipment followed by intracellular analysis of Kat expression with the FLoid Cell Imaging Station. As expected, given the potential scattered insertion of the *Kat* expression cassette in the genome of the recipients, different mutants displayed different levels of fluorescence. Of 69 mutants evaluated for far-red Kat-fluorescence, 58 exhibited high levels of positive fluorescence (Figure 3).

**Figure 4 -:**
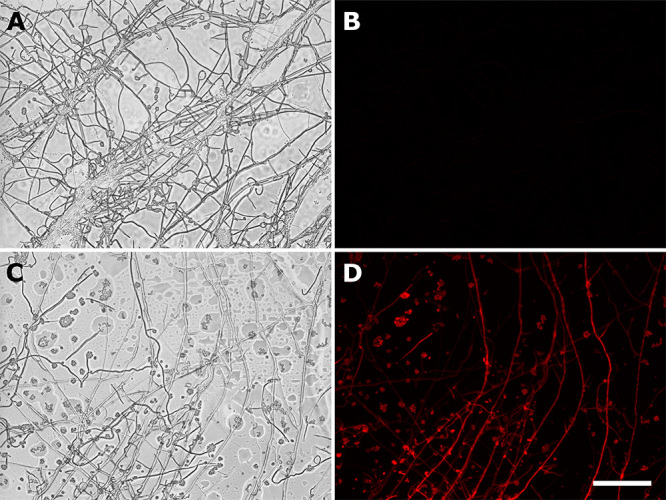
Microscopic evaluation of far-red Kat-fluorescence. Isolated mutants were evaluated for Kat-fluorescence employing the FLoid Cell Imaging Station. **A)** *S. subtropicum* wild-type strain relief phase. **B)** Wild-type red fluorescence. **C)** *S. subtropicum* mutant strain, gpdA-Kat-Sur (+), relief phase. **D)** gpdA-Kat-Sur (+) red fluorescence. Scale bar, 100 μm, for all images.

To rule out that the observed fluorescence could be due to the presence of residual *A. tumefaciens* cells, we also evaluated the generated mutants by fluorescence microscopy analysis. As expected, due to the use of a strong and constitutive promoter, the Kat fluorescence was detected in both mycelia and spores of a representative transformant (Figure 4). Noteworthy, while we could detect fluorescence in fungal cells, the bacterial strain harboring the plasmid pPZP201BK::SUR::gpdA::Kat::TrpC has no clear phenotype (Figure S2). Therefore, besides showing CE resistance, several transformants also carry the active *Kat* expression cassette, reiterating the success of the developed ATMT method.

**Figure 5 -:**
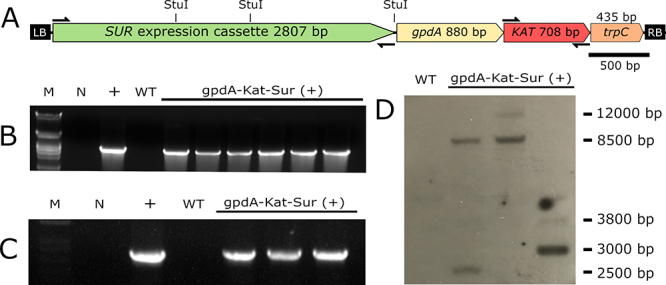
Evaluation of the *SUR* gene and *Kat* expression cassette stability in the *S. subtropicum* mutants. Selected *S. subtropicum* mutants with the brightest far-red Kat-fluorescence were cultivated for five generations in M Cc without CE to evaluate the mitotic stability. **A)** The *Kat* gene expression cassette (gpdA::Kat::TrpC) was cloned next to the *SUR* expression cassette (as displayed) in the plasmid pPZP201BK::SUR, to generate the plasmid pPZP201BK::SUR::gpdA::Kat::TrpC. **B)** The PCR results amplifying the *SUR* gene (~2800 bp). **C)** The PCR results amplifying the *KAT* CDS (~700 bp). **D)** The Southern blotting results employing the *KAT* CDS as a probe. M - DNA Ladder; N - Negative control (without DNA); + - pPZP201BK::SUR::gpdA::Kat::TrpC plasmid; WT - *S. subtropicum* wild-type strain; gpdA-Kat-Sur (+) - *S. subtropicum* mutant strains harboring the *Kat* gene expression cassette and the *SUR* gene integrated into the genome.

Six mutants, which displayed the brightest far-red Kat-fluorescence, were chosen to assess mitotic stability. These mutants were cultivated in MCc medium without selection of CE during five generations. All mutants maintained the fluorescence even in MCc (data not shown). Moreover, the DNA of these mutants was extracted, and PCR was employed to evaluate the presence of the *SUR* gene and the *Kat* expression cassette (Figure 5A). All mutants presented amplification for the *SUR* gene (~ 2800 bp; Figure 5B). Additionally, three selected mutants were also PCR-assayed for the *KAT* CDS (Figure 5C), and the same mutants were inspected by Southern blotting (Figure 5D). Therefore, even with the brightest far-red Kat-fluorescence (i. e., pointing for putative stronger expression/ higher protein content) the insertions in the *S. subtropicum* genome were stable for at least five generations. Notably, the mutants that displayed the brightest far-red Kat-fluorescence presented at least two insertions of the *Kat* expression cassette in the genome (Figure 5D). The stability of the construct, together with the usefulness of the macro- and micro-visualization of the Kat fluorescence (Figures 3 and 4, respectively) can be valuable for genetic studies in *Simplicillium*.

## Discussion

Methods for the transformation of fungi are basic to understand molecular aspects of these species. In addition, the impact of genetic manipulation has revolutionized modern biotechnology, and filamentous fungi are a well-established and important source of enzymes and bioactive molecules (Idnurm and Meyer, 2014; Khan *et al*., 2016). The implementation of *A. tumefaciens* as a tool remodeled several approaches to discover and understand gene function in many fungal species (Idnurm *et al*., 2017). The *Simplicillium* genus has drawn increase scientific interest and a method for genetic transformation was still absent.

Different strategies have been used for the transformation of filamentous fungi (Ruiz-Díez, 2002; Li *et al*., 2017). These methods range from shock-wave-mediated transformation to protoplast-mediated transformation and ATMT (Li *et al*., 2017). Although laborious, the ATMT method has been explored in several organisms, being, usually, the first method standardized for fungi that lack established genetic transformation strategies (Idnurm *et al*., 2017). Although there are several ATMT protocols established, the co-cultivation of *A. tumefaciens*-fungus is a central step. Small variations in *A. tumefaciens* strains, membranes employed/solid support, as well as mutant plating and selection, are crucial for transformation success/failure and efficiency. The robust protocol developed for ATMT of *A. awamori* has been previously adapted for hard-to-transform fungi (Michielse *et al*., 2008), as *Paracoccidioides* spp. (Almeida *et al*., 2007; Menino *et al*., 2012; Bailão *et al*., 2014; Fernandes *et al*., 2017; Nora *et al*., 2019; Silva *et al*., 2020), and, for that reason, this method was chosen.

As entomopathogenic species, future studies in *Simplicillium* spp. can focus on the heterologous expression of toxins and virulence determinants. This approach has been successfully implemented in *Metarhizium* spp. (Wang and St Leger, 2007; Bilgo *et al*., 2017). Recently, a semifield trial of a transgenic *Metarhizium pingshaense* expression insect-specific toxins has shown high efficiency (Lovett *et al*., 2019). Similarly, genetic engineering can improve the biosorption capacity of *Simplicillium* spp. The expression of cell wall metal-binding chimeric ligands increased Cd^2+^ and Zn^2+^ recovery in *Saccharomyces cerevisiae* (Vinopal *et al*., 2007). Furthermore, T-DNA libraries can be useful for the characterization of *S. subtropicum* genes of overall importance, enrolled in heavy metal tolerance/assimilation and virulence determinants (Zhao *et al*., 2014).

A diverse array of secondary metabolites has been isolated from *Simplicillium* species. In recent years, genome mining of secondary metabolite biosynthetic gene clusters (BGCs), coupled with knockout strains, overexpression of transcription factors, and heterologous expression of BGCs have been largely employed for the discovery of biosynthetic pathways (Gilchrist *et al*., 2018). Although genomes from *Simplicillium* spp. are not yet available, that should be one of the main goals going forward. Besides, secondary metabolites, the genome sequencing of these species, coupled with the standardized ATMT method, can reveal important aspects of the life and infection cycle of fungi from the *Simplicillium* genus.

## Supplementary material

Supplementary Data S1 - Alignment of ITS sequences used to generate de *Simplicillium* species tree.

Table S1 **-** Sequences of primers used in this work.

Figure S1 **-** pPZP201BK::SUR::gpdA::Kat::TrpC plasmid map.

Supplementary Data S2 **-** pPZP201BK::SUR::gpdA::Kat::TrpC plasmid sequence.

Figure S2 **-** Confirmation of fluorescence detection in the fungal transformants.
