# Supplementary material for: An efficient Agrobacterium tumefaciens-mediated transformation method for Simplicillium subtropicum (Hypocreales: Cordycipitaceae)
Source: Genet Mol Biol. 2021 Oct 1;44(3):e20210073. doi: 10.1590/1678-4685-GMB-2021-0073 (PMC8489804; doi:10.1590/1678-4685-GMB-2021-0073)
Supplement: Supplementary Data S1 - [file 1415-4757-GMB-44-3-e20210073-s1.pdf]

Genetics and Molecular Biology
https://doi.org/10.1590/1678-4685-GMB-2021-0073

Supplementary Material to "An efficient *Agrobacterium tumefaciens*-mediated transformation method for *Simplicillium subtropicum* (Hypocreales: Cordycipitaceae)"

Supplementary Data S1 - Alignment of ITS sequences used to generate de *Simplicillium* species tree.

Supplementary Material 1

>NR_119828.1 [Bionectria] vesiculosa HMAS 183151 ITS region; from TYPE material

----------------------TTCCGTTGGTGAACCAGCGTATCATTACT-GAGTA-CAACTCCCACCCA-TGTGAAC--
ACC------A-GTT-GCTTCGGCG--GTC--CCG--------------CGGATCCAGGCG-C-------CAACTC---------------
---------------CGAGT-CTGAG----------
TAATCAAAACTTTCAACAACGGATCTCTTGGTTCTGGCATCGATGAAGAACGCAGCGAAATGCGAAAA
GTAATGTGAATTGCAGAATTCAGTGAATCATCGAATCTTTGAACGCACATTGCGCCCGCCAGCATTCTG
GCGGGCATGCCTGTCTGAGCGTCATTTCAACCCTCACT----TT-GGCGTTGGGGATCGGC------------------
----
CCGGCCCCGAAATATAGTGGCGGACCCGCTGTGGCCTCCTCTGCGAAGTAG----CCGCACCGG-A-A-
GCAGCGAGCCCCTGCGTTAAACCCCCCAAT---------
AAGGTTGACCTCAGATCAGGTAGGAATACCCGCTGAACTTAA---

>NR_077207.1 Trichoderma atroviride NBRC 101776 ITS region; from synonym TYPE material

----------------------TTCCGTTGGTGAACCAGCGGATCATTACC-GAGTA-CAACTCCCACCCAATGTGAAC-
-ACC------ATGTT-GCCTCGGCG--GTCACCCG--------CGCA---CGGAACCAGGCG-C-GACC--AAACTC-----
---GTCCCCTCGCGGACGTTATTTC-------CTGAG--------
ATGAATCAAAACTTTCAACAACGGATCTCTTGGTTCTGGCATCGATGAAGAACGCAGCGAAATGCGATA
AGTAATGTGAATTGCAGAATTCAGTGAATCATCGAATCTTTGAACGCACATTGCGCCCGCCAGTATTCT
GGCGGGCATGCCTGTCCGAGCGTCATTTCAACCCTCGAA----GTCGGCGTTGGGGA----C-----------------
--------------------------------GAG---------------------
GATCCCGGCCCCGAAATACAGTGGCGGTCTCGCCGCAGCCTCTCCTGCGCAGTAGTGC-TCGCACCGG-
G-GCGCGGCGCGTCCACGCCGTAAAACACCCAAC---------
AATGTTGACCTCGGATCAGGTAGGAATACCCGCTGAACTTAA---

>AJ292397.1 Verticillium chlamydosporium var. chlamydosporium partial 18S rRNA gene, internal transcribed spacer 1 (ITS1), 5.8S rRNA gene, internal transcribed spacer 2 (ITS2) and partial 28S rRNA gene, strain CBS 103.65

---ATTACC-GAGTT-CAACTCCCACCCCATGTGAACTTACC--
TTACACGTT-GCTTCGGCG--GTCTCCCG--------------CGGAACCAGGCGGC-------AAACTC----------------
------------G------CTGAGTGG-----
ATGAATCAAAACTTTCAACAACGGATCTCTTGGTTCTGGCATCGATGAAGAACGCAGCGAAATGCGATA
AGTAATGTGAATTGCAGAATTCAGTGAATCATCGAATCTTTGAACGCACATTGCGCCCGCCAGTATTCT
GGCGGGCATGCCTGTTCGAGCGTCATTTCAACCCTCAAG----TT-
GGTGTTGGGGACCGGCGAGTACAGAGGCTTTGGGGACTTGTCCCCCTTCC-------------------------------
-----------CCGCCCCCGAAATGAATTGGCGGTCTCGTCGCGGCCTCCTCTGCGTAGTAG----
TCGCATCAG-G-GCGCGGCGCGGCCACTCCGTAAAACGCCCAAC----
TTTTAAGAGTTGACCTCGAATCAGGTAGGAATACCCG--

>AB603997.1 Simplicillium cylindrosporum genes for 18S rRNA, ITS1, 5.8S rRNA, ITS2, 28S rRNA, partial and complete sequence, strain: JCM 18171

----------------------TCCGTAGGTGAACCTGCGGATCATTATC-GAGTATCAACTCCC-CCCTATGTGAAC--
---CTTTA--TGTT-GCTTCGGCG--GTCGC---T-----GC-------CGGGACCACGCG-T-------AAACTC--CGAAA--
-------------------------TCTGAGTGG---
CAATGAATCAAAACTTTCAACAACGGATCTCTTGGTTCTGGCATCGATGAAGAACGCAGCGAAATGCG
ATAAGTAATGTGAATTGCAGAATTCAGTGAATCATCGAATCTTTGAACGCACATTGCGCCCGCCAGCAT
TCTGGCGGGCATGCCTGTTCGAGCGTCATTTCAACCCTCGAGGAGATC-GGTGTTGGGACCCGGC-------
--GACTTCTG----------------CCGGCCCCGAAATTCAGTGGCGG-
CCCGTTGCGGCGACCTCTGCGTAGTAA----TCGCACTGG-G-
CAGCATCGCGGCCACGCCGTAAAACCCCCGAC---------
AAGGTTGACCTCGAATCAGGTAGGACTACCCGCTGAACTTAAGCATATCAAT--CGGAGG----------------
----

---------------------------------------A

>AB603998.1 Simplicillium cylindrosporum genes for 18S rRNA, ITS1, 5.8S rRNA, ITS2, 28S rRNA, partial and complete sequence, strain: JCM 18172

-------------------------------GAACCTGCGGATCATTATC-GAGTATCAACTCCC-CCCTATGTGAAC-----
CTTTA--TGTT-GCTTCGGCG--GTCGC---T-----GC-------CGGGACCACGCG-T-------AAACTC--CGAAA-----
----------------------TCTGAGTGG---
CAATGAATCAAAACTTTCAACAACGGATCTCTTGGTTCTGGCATCGATGAAGAACGCAGCGAAATGCG
ATAAGTAATGTGAATTGCAGAATTCAGTGAATCATCGAATCTTTGAACGCACATTGCGCCCGCCAGCAT

TCTGGCGGGCATGCCTGTTCGAGCGTCATTTCAACCCTCGAGGAGATC-GGTGTTGGGACCCGGC-------
--GACTTCTG----------------CCGGCCCCGAAATTCAGTGGCGG-
CCCGTTGCGGCGACCTCTGCGTAGTAA----TCGCACTGG-G-
CAGCATCGCGGCCACGCCGTAAAACCCCCGAC---------
AAGGTTGACCTCGAATCAGGTAGGACTACCCGCTGAACTTAAGCATATCAAT--CGGAGG----------------
----

>AB604006.1 Simplicillium cylindrosporum genes for 18S rRNA, ITS1, 5.8S rRNA, ITS2, 28S rRNA, partial and complete sequence, strain: JCM 18175

----------------------TCCGTAGGTGAACCTGCGGATCATTATC-GAGTATCAACTCCC-CCCTATGTGAAC--
---CTTTA--TGTT-GCTTCGGCG--GTCGC---T-----GC-------CGGGACCACGCG-T-------AAACTC--CGAAA--
-------------------------TCTGAGTGG---
CAATGAATCAAAACTTTCAACAACGGATCTCTTGGTTCTGGCATCGATGAAGAACGCAGCGAAATGCG
ATAAGTAATGTGAATTGCAGAATTCAGTGAATCATCGAATCTTTGAACGCACATTGCGCCCGCCAGCAT
TCTGGCGGGCATGCCTGTTCGAGCGTCATTTCAACCCTCGAGGAGATC-GGTGTTGGGACCCGGC-------
--GACTTCTG----------------CCGGCCCCGAAATTCAGTGGCGG-
CCCGTTGCGGCGACCTCTGCGTAGTAA----TCGCACTGG-G-
CAGCATCGCGGCCACGCCGTAAAACCCCCGAC---------
AAGGTTGACCTCGAATCAGGTAGGACTACCCGCTGAACTTAAGCATATCAAT-ACGGAGG---------------
----

--A

>AB604005.1 Simplicillium cylindrosporum genes for 18S rRNA, ITS1, 5.8S rRNA, ITS2, 28S rRNA, partial and complete sequence, strain: JCM 18174

----------------------TCCGTAGGTGAACCTGCGGATCATTATC-GAGTATCAACTCCC-CCCTATGTGAAC--
---CTTTA--TGTT-GCTTCGGCG--GTCGC---T-----GC-------CGGGACCACGCG-T-------AAACTC--CGAAA--
-------------------------TCTGAGTGG---
CAATGAATCAAAACTTTCAACAACGGATCTCTTGGTTCTGGCATCGATGAAGAACGCAGCGAAATGCG
ATAAGTAATGTGAATTGCAGAATTCAGTGAATCATCGAATCTTTGAACGCACATTGCGCCCGCCAGCAT
TCTGGCGGGCATGCCTGTTCGAGCGTCATTTCAACCCTCGAGGAGATC-GGTGTTGGGACCCGGC-------
--GACTTCTG----------------CCGGCCCCGAAATTCAGTGGCGG-
CCCGTTGCGGCGACCTCTGCGTAGTAA----TCGCACTGG-G-
CAGCATCGCGGCCACGCCGTAAAACCCCCGAC---------
AAGGTTGACCTCGAATCAGGTAGGACTACCCGCTGAACTTAAGCATATCAAT-ACGGAGG---------------
----

--A

>AB603994.1 Simplicillium cylindrosporum genes for 18S rRNA, ITS1, 5.8S rRNA, ITS2, 28S rRNA, partial and complete sequence, strain: JCM 18170

----------------------------------GAACCTGCGGATCATTATC-GAGTATCAACTCCC-CCCTATGTGAAC-----
CTTTA--TGTT-GCTTCGGCG--GTCGC---T-----GC-------CGGGACCACGCG-T-------AAACTC--CGAAA-----
-------------------------TCTGAGTGG---
CAATGAATCAAAACTTTCAACAACGGATCTCTTGGTTCTGGCATCGATGAAGAACGCAGCGAAATGCG
ATAAGTAATGTGAATTGCAGAATTCAGTGAATCATCGAATCTTTGAACGCACATTGCGCCCGCCAGCAT
TCTGGCGGGCATGCCTGTTCGAGCGTCATTTCAACCCTCGAGGAGATC-GGTGTTGGGACCCGGC-------
--GACTTCTG-----------------CCGGCCCCGAAATTCAGTGGCGG-
CCCGTTGCGGCGACCTCTGCGTAGTAA----TCGCACTGG-G-
CAGCATCGCGGCCACGCCGTAAAACCCCCGAC---------
AAGGTTGACCTCGAATCAGGTAGGACTACCCGCTGAACTTAAGCATATCAAT-ACGGAGG----------------
----

---A

>AB603999.1 Simplicillium cylindrosporum genes for 18S rRNA, ITS1, 5.8S rRNA, ITS2, 28S rRNA, partial and complete sequence, strain: JCM 18173

---------------------------CCGTAGGGTAACCTGCGGATCATTATC-GAGTATCAACTCCC-CCCTATGTGAAC---
--CTTTA--TGTT-GCTTCGGCG--GTCGC---T-----GC-------CGGGACCACGCG-T-------AAACTC--CGAAA---
---------------------------TCTGAGTGG---
CAATGAATCAAAACTTTCAACAACGGATCTCTTGGTTCTGGCATCGATGAAGAACGCAGCGAAATGCG
ATAAGTAATGTGAATTGCAGAATTCAGTGAATCATCGAATCTTTGAACGCACATTGCGCCCGCCAGCAT
TCTGGCGGGCATGCCTGTTCGAGCGTCATTTCAACCCTCGAGGAGATC-GGTGTTGGGACCCGGC-------
--GACTTCTG-----------------CCGGCCCCGAAATTCAGTGGCGG-
CCCGTTGCGGCGACCTCTGCGTAGTAA----TCGCACTGG-G-
CAGCATCGCGGCCACGCCGTAAAACCCCCGAC---------
AAGGTTGACCTCGAATCAGGTAGGACTACCCGCTGAACTTAAGCATATCAAT--CGGAGG-----------------
----

--A

>AB603989.1 Simplicillium cylindrosporum genes for 18S rRNA, ITS1, 5.8S rRNA, ITS2, 28S rRNA, partial and complete sequence, strain: JCM 18169

--------------------------------------ACCTGCGGATCATTATC-GAGTATCAACTCCC-CCCTATGTGAAC-----
CTTTA--TGTT-GCTTCGGCG--GTCGC---T-----GC-------CGGGACCACGCG-T-------AAACTC--CGAAA-----
-------------------------TCTGAGTGG---
CAATGAATCAAAACTTTCAACAACGGATCTCTTGGTTCTGGCATCGATGAAGAACGCAGCGAAATGCG
ATAAGTAATGTGAATTGCAGAATTCAGTGAATCATCGAATCTTTGAACGCACATTGCGCCCGCCAGCAT
TCTGGCGGGCATGCCTGTTCGAGCGTCATTTCAACCCTCGAGGAGATC-GGTGTTGGGACCCGGC-------
--GACTTCTG-----------------CCGGCCCCGAAATTCAGTGGCGG-
CCCGTTGCGGCGACCTCTGCGTAGTAA----TCGCACTGG-G-
CAGCATCGCGGCCACGCCGTAAAACCCCCGAC---------

AAGGTTGACCTCGAATCAGGTAGGACTACCCGCTGAACTTAAGCATATCAAT---------------------------
----

>AB603991.1 Simplicillium minatense genes for 18S rRNA, ITS1, 5.8S rRNA, ITS2, 28S rRNA, partial and complete sequence, strain: JCM 18177

----------------------------GAACCTGCGGATCATTATC-GAGTATCAACTCCC-CCCTTTGTGAAC-----
CTTTA--TGTT-GCTTCGGCG--GTCGC---T-----GC-------CGGGACCACGCG-T-------AAACTC--CGAAA-----
---------------------TCTGAGTGG---
CAATGAATCAAAACTTTCAACAACGGATCTCTTGGTTCTGGCATCGATGAAGAACGCAGCGAAATGCG
ATAAGTAATGTGAATTGCAGAATTCAGTGAATCATCGAATCTTTGAACGCACATTGCGCCCGCCAGCAT
TCTGGCGGGCATGCCTGTTCGAGCGTCATTTCAACCCTCGAGGAGATC-GGTGTTGGGACCCGGC-------
---GACTTCTG---------------CCGGCCCCGAAATTCAGTGGCGG-
CCCGTTGCGGCGACCTCTGCGTAGTAA----TCGCACTGG-G-
CAGCATCGTGGCCACGCCGTAAAACCCCCGAC--------
AAGGTTGACCTCGAATCAGGTAGGACTACCCGCTGAACTTAAGCATATCAAT--CGGAGG---------------
----

-----------------------------------A

>AB603992.1 Simplicillium minatense genes for 18S rRNA, ITS1, 5.8S rRNA, ITS2, 28S rRNA, partial and complete sequence, strain: JCM 18176

----------------------------GAACCTGCGGATCATTATC-GAGTATCAACTCCC-CCCTTTGTGAAC-----
CTTTA--TGTT-GCTTCGGCG--GTCGC---T-----GC-------CGGGACCACGCG-T-------AAACTC--CGAAA-----
---------------------TCTGAGTGG---
CAATGAATCAAAACTTTCAACAACGGATCTCTTGGTTCTGGCATCGATGAAGAACGCAGCGAAATGCG
ATAAGTAATGTGAATTGCAGAATTCAGTGAATCATCGAATCTTTGAACGCACATTGCGCCCGCCAGCAT
TCTGGCGGGCATGCCTGTTCGAGCGTCATTTCAACCCTCGAGGAGATC-GGTGTTGGGACCCGGC-------
---GACTTCTG---------------CCGGCCCCGAAATTCAGTGGCGG-
CCCGTTGCGGCGACCTCTGCGTAGTAA----TCGCACTGG-G-
CAGCATCGTGGCCACGCCGTAAAACCCCCGAC-T------
AAGGTTGACCTCGAATCAGGTAGGACTACCCGCTGAACTTAAGCATATCAAT--CGGAGG---------------
----

>AB603993.1 Simplicillium minatense genes for 18S rRNA, ITS1, 5.8S rRNA, ITS2, 28S rRNA, partial and complete sequence, strain: JCM 18178

----------------------------GAACCTGCGGATCATTATC-GAGTATCAACTCCC-CCCTTTGTGAAC-----
CTTTA--TGTT-GCTTCGGCG--GTCGC---T-----GC-------CGGGACCACGCG-T-------AAACTC--CGAAA-----
---------------------TCTGAGTGG---

CAATGAATCAAAACTTTCAACAACGGATCTCTTGGTTCTGGCATCGATGAAGAACGCAGCGAAATGCG
ATAAGTAATGTGAATTGCAGAATTCAGTGAATCATCGAATCTTTGAACGCACATTGCGCCCGCCAGCAT
TCTGGCGGGCATGCCTGTTCGAGCGTCATTTCAACCCTCGAGGAGATC-GGTGTTGGGACCCGGC-------
---GACTTCTG-----------------CCGGCCCCGAAATTCAGTGGCGG-
CCCGTTGCGGCGACCTCTGCGTAGTAA----TCGCACTGG-G-
CAGCATCGTGGCCACGCCGTAAAACCCCCGAC-T-------
AAGGTTGACCTCGAATCAGGTAGGACTACCCGCTGAACTTAAGCATATCAAT--CGGAGG-----------------
----

--T

>AJ292396.1 Cephalosporium lanoso-niveum partial 18S rRNA gene, internal transcribed spacer 1 (ITS1), 5.8S rRNA gene, internal transcribed spacer 2 (ITS2) and partial 28S rRNA gene, strain CBS 704.86

--ATTATC-GAGTATCAACTCCC-CCCTATGTGAAC-----CTTTA--
TGTT-GCTTCGGCG--GTCGC---T-----GC-------CGGGACCACGCG-T-------AAACTC--CGAAA---------------
--------------TCTGAGTGG---
CAATGAATCAAAACTTTCAACAACGGATCTCTTGGTTCTGGCATCGATGAAGAACGCAGCGAAATGCG
ATAAGTAATGTGAATTGCAGAATTCAGTGAATCATCGAATCTTTGAACGCACATTGCGCCCGCCAGCAT
TCTGGCGGGCATGCCTGTTCGAGCGTCATTTCAACCCTCGAGGAGATC-GGTGTTGGGACCCGGC-------
---GACTCTTG-----------------CCGGCCCCGAAATTCAGTGGCGG-
CCCGTTGCGGCGACCTCTGCGTAGTAA----TCGCACCGGTA-
CAGCATCGTGGCCACGCCGTAAAACCCCCGAC---------
AAGGTTGACCTCGAATCAGGTAGGAATACCCG--

>EF641862.1 Simplicillium lanosoniveum strain CBS 962.72 18S ribosomal RNA gene, partial sequence; internal transcribed spacer 1, 5.8S ribosomal RNA gene, and internal transcribed spacer 2, complete sequence; and 28S ribosomal RNA gene, partial sequence

---ACTATTATCTGAGTATCAACTCCC-CCCTATGTGAAC-----CTTTA--
TGTTTGCTTCGGCG--GTCGC---T-----GC-------CGGGACCACGCG-T-------AAACTC--CGAAA--------------
---------------TCTGAGTGG---
CAATGAATCAAAACTTTCAACAACGGATCTCTTGGTTCTGGCATCGATGAAGAACGCAGCGAAATGCG
ATAAGTAATGTGAATTGCAGAATTCAGTGAATCATCGAATCTTTGAACGCACATTGCGCCCGCCAGCAT
TCTGGCGGGCATGCCTGTTCGAGCGTCATTTCAACCCTCGAGGAGATC-GGTGTTGGGACCCGGC-------
---GACTCTTG-----------------CCGGCCCCGAAATTCAGTGGCGG-
CCCGTTGCGGCGACCTCTGCGTAGTAA----TCGCACCGGTA-
CAGCATCGTGGCCACGCCGTAAAACCCCCGAC---------AAGGTTGACCTCGAATCAGGTA-
GACTACCCGCTGA-------CAT---

>AB604001.1 Simplicillium subtropicum genes for 18S rRNA, ITS1, 5.8S rRNA, ITS2, 28S rRNA, partial and complete sequence, strain: JCM 18183

----------------------TCCGTAGGTGAACCTGCGGATCATTATC-GAGTATCAACTCCC-CCCTATGTGAAC--
---CTTTA--TGTT-GCTTCGGCG--GTCGC---T-----GC-------CGGGACCACGCG-T-------AAACTC--CGAAA--
--------------------------TCTGAGTGG---
CAATGAATCAAAACTTTCAACAACGGATCTCTTGGTTCTGGCATCGATGAAGAACGCAGCGAAATGCG
ATAAGTAATGTGAATTGCAGAATTCAGTGAATCATCGAATCTTTGAACGCACATTGCGCCCGCCAGCAT
TCTGGCGGGCATGCCTGTTCGAGCGTCATTTCAACCCTCGAGGAGATC-GGTGTTGGGACCCGGC-------
---GACTCACG----------------CCGGCCCCGAAATTCAGTGGCGG-
CCCGTTGCGGCGACCTCTGCGTAGTAA----TCGCACTGG-G-
CAGCAGCGCGGCCACGCCGTAAAACCCCCGAC---------
AAGGTTGACCTCGAATCAGGTAGGACTACCCGCTGAACTTAAGCATATCAAT--CGGAGG----------------
----

>AB603996.1 Simplicillium subtropicum genes for 18S rRNA, ITS1, 5.8S rRNA, ITS2, 28S rRNA, partial and complete sequence, strain: JCM 18182

----------------------TCCGTAGGTGAACCTGCGGATCATTATC-GAGTATCAACTCCC-CCCTATGTGAAC--
---CTTTA--TGTT-GCTTCGGCG--GTCGC---T-----GC-------CGGGACCACGCG-T-------AAACTC--CGAAA--
--------------------------TCTGAGTGG---
CAATGAATCAAAACTTTCAACAACGGATCTCTTGGTTCTGGCATCGATGAAGAACGCAGCGAAATGCG
ATAAGTAATGTGAATTGCAGAATTCAGTGAATCATCGAATCTTTGAACGCACATTGCGCCCGCCAGCAT
TCTGGCGGGCATGCCTGTTCGAGCGTCATTTCAACCCTCGAGGAGATC-GGTGTTGGGACCCGGC-------
---GACTCACG----------------CCGGCCCCGAAATTCAGTGGCGG-
CCCGTTGCGGCGACCTCTGCGTAGTAA----TCGCACTGG-G-
CAGCAGCGCGGCCACGCCGTAAAACCCCCGAC---------
AAGGTTGACCTCGAATCAGGTAGGACTACCCGCTGAACTTAAGCATATCAAT--CGGAGG----------------
----

---A

>AB603995.1 Simplicillium subtropicum genes for 18S rRNA, ITS1, 5.8S rRNA, ITS2, 28S rRNA, partial and complete sequence, strain: JCM 18181

--------------------------------GAACCTGCGGATCATTATC-GAGTATCAACTCCC-CCCTATGTGAAC-----
CTTTA--TGTT-GCTTCGGCG--GTCGC---T-----GC-------CGGGACCACGCG-T-------AAACTC--CGAAA-----
----------------------TCTGAGTGG---
CAATGAATCAAAACTTTCAACAACGGATCTCTTGGTTCTGGCATCGATGAAGAACGCAGCGAAATGCG
ATAAGTAATGTGAATTGCAGAATTCAGTGAATCATCGAATCTTTGAACGCACATTGCGCCCGCCAGCAT
TCTGGCGGGCATGCCTGTTCGAGCGTCATTTCAACCCTCGAGGAGATC-GGTGTTGGGACCCGGC-------
---GACTCACG----------------CCGGCCCCGAAATTCAGTGGCGG-

CCCGTTGCGGCGACCTCTGCGTAGTAA----TCGCACTGG-G-
CAGCAGCGCGGCCACGCCGTAAAACCCCCGAC---------
AAGGTTGACCTCGAATCAGGTAGGACTACCCGCTGAACTTAAGCATATCAAT--CGGAGG------------------
----

--A

>SIMP

TGGAAGTAAAAGTCGTAACAAGGTTCCGTTGGTGAACCAGCGGATCATTATC-GAGTATCAACTCCC-
CCCTATGTGAAC-----CTTTA--TGTT-GCTTCGGCG--GTCGC---TC----GC-------CGGGACCACGCG-T-------
AAACTC--CGAAA-------------------------------TCTGAGTGG---
CAATGAATCAAAACTTTCAACAACGGATCTCTTGGTTCTGGCATCGATGAAGAACGCAGCGAAATGCG
ATAAGTAATGTGAATTGCAGAATTCAGTGAATCATCGAATCTTTGAACGCACATTGCGCCCGCCAGCAT
TCTGGCGGGCATGCCTGTTCGAGCGTCATTTCAACCCTCGAGGAGATC-GGTGTTGGGATCCGGC-------
--GGACTCACG-T----------------CCGGTCCCGAAATTCAGTGGCGG-
CCCGTTGCGGCGACCTCTGCGTAGTAA----TCGCACTGG-G-
CAGCAGCGCGGCCACGCCGTAAAACCCCCGAC---------
AAGGTTGACCTCGAATCAGGTAGGACTACCCGCTGAACTTAAGCATATCAATN-CGGAGG-----------------
----

--A

>AB603990.1 Simplicillium subtropicum genes for 18S rRNA, ITS1, 5.8S rRNA, ITS2, 28S rRNA, partial and complete sequence, strain: JCM 18180

---------------------------------------CTGCGGATCATTATC-GAGTATCAACTCCC-CCCTATGTGAAC-----
CTTTA--TGTT-GCTTCGGCG--GTCGC---T-----GC-------CGGGACCACGCG-T-------AAACTC--CGAAA-----
--------------------------TCTGAGTGG---
CAATGAATCAAAACTTTCAACAACGGATCTCTTGGTTCTGGCATCGATGAAGAACGCAGCGAAATGCG
ATAAGTAATGTGAATTGCAGAATTCAGTGAATCATCGAATCTTTGAACGCACATTGCGCCCGCCAGCAT
TCTGGCGGGCATGCCTGTTCGAGCGTCATTTCAACCCTCGAGGAGATC-GGTGTTGGGACCCGGC-------
---GACTCACG------------------CCGGCCCCGAAATTCAGTGGCGG-
CCCGTTGCGGCGACCTCTGCGTAGTAA----TCGCACTGG-G-
CAGCAGCGCGGCCACGCCGTAAAACCCCCGAC---------
AAGGTTGACCTCGAATCAGGTAGGACTACCCGCTGAACTTAAGCATATCAAT---------------------------------
----

>AB604000.1 Simplicillium obclavatum genes for 18S rRNA, ITS1, 5.8S rRNA, ITS2, 28S rRNA, partial and complete sequence, strain: JCM 18179

--------------------------TCCGTAGGTGAACCTGCGGATCATTATC-GAGTATCAACTCCC-CCCTTTGTGAAC---
--CTTTA--TGTT-GCTTCGGCG--GACGC---T-----GC-------CGGGACCACGCG-C-------AAACTC--CGAAA--

---------------------------TCTGAGTGG--
ACAATGAATCAAAACTTTCAACAACGGATCTCTTGGTTCTGGCATCGATGAAGAACGCAGCGAAATGC
GATAAGTAATGTGAATTGCAGAATTCAGTGAATCATCGAATCTTTGAACGCACATTGCGCCCGCCAGCA
TTCTGGCGGGCATGCCTGTTCGAGCGTCATTTCAACCCTCGAGGAGATC-GGTGTTGGGACCCGGC------
---GACTTTAGT----------------CCGGTCCTGAAATTCAGTGGCGG-
CCCGTTGCGGCGACCTCTGCGTAGTAA----TCGCACTGG-G-
CAGCAGCGCGGCCACGCCGTAAAACCCCCGAC---T-----
AAGGTTGACCTCGAATCAGGTAGGACTACCCGCTGAACTTAAGCATATCAAT-------------------------------
----

>AJ292394.1 Acremonium obclavatum partial 18S rRNA gene, internal transcribed spacer 1 (ITS1), 5.8S rRNA gene, internal transcribed spacer 2 (ITS2) and partial 28S rRNA gene, strain CBS 311.74

--ATTATC-GAGTATCAACTCCC-CCCTTTGTGAAC-----CTTTA--
TGTT-GCTTCGGCG--GACGC---T-----GC-------CGGGACCACGCG-C-------AAACTC--CGAAA---------------
--------------TCTGAGTGG---
CAATGAATCAAAACTTTCAACAACGGATCTCTTGGTTCTGGCATCGATGAAGAACGCAGCGAAATGCG
ATAAGTAATGTGAATTGCAGAATTCAGTGAATCATCGAATCTTTGAACGCACATTGCGCCCGCCAGCAT
TCTGGCGGGCATGCCTGTTCGAGCGTCATTTCAACCCTCGAGGAGATC-GGTGTTGGGACCCGGC-------
--GACTTTAGT----------------CCGGTCCCGAAATTCAGTGGCGG-
CCCGTTGCGGCGACCTCTGCGTAGTAA----TCGCACTGG-G-
CAGCAGCGCGGCCACGCCGTAAAACCCCCGAC---T-----
AAGGTTGACCTCGAATCAGGTAGGACTACCCG--

>AB604002.1 Simplicillium aogashimaense genes for 18S rRNA, ITS1, 5.8S rRNA, ITS2, 28S rRNA, partial and complete sequence, strain: JCM 18167

---------------------------------GAACCTGCGGATCATTATC-GAGTA-CAACTCCC-CCCTATGTGAAC-----
CTTTA--TGTT-GCTTCGGCG--GTCGC---------GC-------CGGGACCACGCG-C-------AAACTC--CGAAA-----
------------------------TCTGAGTGG---
CAATGAATCAAAACTTTCAACAACGGATCTCTTGGTTCTGGCATCGATGAAGAACGCAGCGAAATGCG
ATAAGTAATGTGAATTGCAGAATTCAGTGAATCATCGAATCTTTGAACGCACATTGCGCCCGCCAGCAT
TCTGGCGGGCATGCCTGTTCGAGCGTCATTTCAACCCTCGAGGAGATC-GGTGTTGGGACCCGGC-------
---------------------------AGCGGACCCAG-----------------------------------
CCGGCCCCGAAATTCAGTGGCGG-CCCGTTGCGGCGACCTCTGCGTAGTAA----TCGCAGTGG-A-
GAGCAGCGCGGCCACGCCGTAAAACCCCCGAC---------
AAGGTTGACCTCGAATCAGGTAGGACTACCCGCTGAACTTAAGCATATCAAT-------------------------------
----

>AB604004.1 Simplicillium aogashimaense genes for 18S rRNA, ITS1, 5.8S rRNA, ITS2, 28S rRNA, partial and complete sequence, strain: JCM 18168

-------------------------------ACCTGCGGATCATTATC-GAGTA-CAACTCCC-CCCTATGTGAAC-----
CTTTA--TGTT-GCTTCGGCG--GTCGC---------GC-------CGGGACCACGCG-C-------AAACTC--CGAAA-----
---------------------TCTGAGTGG---
CAATGAATCAAAACTTTCAACAACGGATCTCTTGGTTCTGGCATCGATGAAGAACGCAGCGAAATGCG
ATAAGTAATGTGAATTGCAGAATTCAGTGAATCATCGAATCTTTGAACGCACATTGCGCCCGCCAGCAT
TCTGGCGGGCATGCCTGTTCGAGCGTCATTTCAACCCTCGAGGAGATC-GGTGTTGGGACCCGGC-------
------------------------AGCGGACCCAG--------------------------------
CCGGCCCCGAAATTCAGTGGCGG-CCCGTTGCGGCGACCTCTGCGTAGTAA----TCGCAGTGG-A-
GAGCAGCGCGGCCACGCCGTAAAACCCCCGAC---------
AAGGTTGACCTCGAATCAGGTAGGACTACCCGCTGAACTTAAGCATATCAAT--CGGAGG---------------
----

-------------------------------------A

>AB604003.1 Simplicillium sympodiophorum genes for 18S rRNA, ITS1, 5.8S rRNA, ITS2, 28S rRNA, partial and complete sequence, strain: JCM 18184

---------------------TCCGTAGGTGAACCTGCGGATCATTAAC-GAG-ATCAACTCCCACCCTATGTGAAC--
ACC--TTA--CGTT-GCTTCGGCG-TG-CGC-----TGCTCC----GGGCA----AACGCG-CA------AAACTC--
CGAAA--------------------------TCTGAGTGGA--
CAATGAATCAAAACTTTCAACAACGGATCTCTTGGTTCTGGCATCGATGAAGAACGCAGCAAAATGCGA
TAAGTAATGTGAATTGCAGAATTCAGTGAATCATCGAATCTTTGAACGCACATTGCGCCCGCCAGCATT
CTGGCGGGCATGCCTGTTCGAGCGTCATTTCAACCCTCGACGAGGCC-GGTGTTGGGGCGCGGC--------
---AGCTCGC--------CCGGCTCCGAAATTCAGTGGCGG-
CCCGTTGCGGCGACCTCTGCGTAGTAA---CTCGCACTGG-G-
CAGCAGCGCGGCCACGCCGTAAAACCCCCGAC--T------
AAGGTTGACCTCGAATCAGGTAGGACTACCCGCTGAACTTAAGCATATCAAT--CGGAGG---------------
----

-------------------------------------A

>KU746706.1 Simplicillium calcicola strain LC5586 internal transcribed spacer 1, partial sequence; 5.8S ribosomal RNA gene and internal transcribed spacer 2, complete sequence; and 28S ribosomal RNA gene, partial sequence

--ACTCCC-CCCTTTGTGA-C--ACC--TTA--CGTT-
GCTTCGGCG--G-CGC----------------CGGGTCAACGCC-T------TAAACTC--CGAAA-----------------------
---TCTGAGTGG---
CAATGAATCAAAACTTTCAACAACGGATCTCTTGGTTCTGGCATCGATGAAGAACGCAGCAAAATGCGA
TAAGTAATGTGAATTGCAGAATTCAGTGAATCATCGAATCTTTGAACGCACATTGCGCCCGCCAGCATT
CTGGCGGGCATGCCTGTTCGAGCGTCATTTCAACCCTCGACGAAGCC-GGTGTTGGGGCGCGGC--------

---AGCTCGC--------CCGGCTCCGAAATCTAGTGGCGG-CCCGTTGCGGCGACCTCTGCGTAGTAA----TCGCACTGG-G-CAGCAGCGCGGCCACGCCGTAAAACCCCCGAC---------AAGGTTGACCTCGAATCAGGTAGGACTACCCGCTGAACTTAAGCATATC---

>AB378533.1 Simplicillium lamellicola genes for 18S rRNA, ITS1, 5.8S rRNA, ITS2, 28S rRNA, partial and complete sequence, isolate: KYK00006

-------------------------CGTTGGTGAACCAGCGGATCATTAAC-GAG-ATCAACTCCCACCCTTTGTGAAC--ACC--TTA--CGTT-GCTTCGGCG--G-CGC------------------CAGGTCAACGCG-C-------AAACTC--CGAAA-----------------------------TCTGAGTGGA--CAATGAATCAAAACTTTCAACAACGGATCTCTTGGTTCTGGCATCGATGAAGAACGCAGCAAAATGCGATAAGTAATGTGAATTGCAGAATTCAGTGAATCATCGAATCTTTGAACGCACATTGCGCCCGCCAGCATTCTGGCGGGCATGCCTGTTCGAGCGTCATTTCAACCCTCGACGAAGCC-GGTGTTGGGGCGCGGC---AGCTCGC--------CCGGCTCCGAAATTTAGTGGCGG-CCCGTTGCGGCGACCTCTGCGTAGTAA----TCGCACTGG-G-CAGCAGCGCGGCCACGCCGTAAAACCCCCGAC---------AAGGTTGACCTCGAATCAGGTAGGACTACCCGCTGAACTTAAGCATATCAAT--CGGAGGAAAGAAACCAACAGGGATTGCCCCAGTAACGGCGAGTGAAGCGGCAACAGCTCAAATTTGAAATCTGGCTCCTAGAGTCCGAATTGTAATTTGCAGAGGATGCTTTTGATGCGGTGCCTTCCGAGTTCCCTGGAACGGGACGCCATAGAGGGTGAGAGCCCCGTCTGGTCGGATGCCAAATCTCTGTAAAGCTCCTTCGACGAGTCGAGTAGTTTGGGAATGCTGCTCTAAATGGGAGGTATATGTCTTCTAAAGCTAAATATTGGCCAGAGACCGATAGCGCACAAGTAGAGTGATCGAAAGATGAAAAGCACTTTGAAAAGAGGGTTAAAAAGTACGTGAAATTGTTGAAAGGGAAGCGCCTATGACCAGACTTGGGCCCGGTGAATCATCCAGCGTTCTCGCTGGTGCACTTTGCCGGGCACAGGCCAGCATCAGTTTGCCTTGGGGGAAAAAGGCTTTGGGAACGTAGCTCCTTCGGGAGTGTTATAGACCATTGCATAATACCCTGGGGCGGACTGAGGTTCGCGCATCTGCAAGGATGCTGGCGTAATGGTCATCAG-

>AF108471.1 Simplicillium lamellicola isolate UAMH 2055 internal transcribed spacer 1, complete sequence

---CATTAAC-GAG-ATCAACTCCCACCCTTTGTGAAC--ACC--TTA--CGTT-GCTTCGGCG--G-CGC------------------CAGGTCAACGCG-C-------AAACTC--CGAAA-----------------------------TCTGAGTGGA--CAATGAATCAAAACTTTCAACAACGGATCTCTTGGTTCTGGCATCGATGAAGAACGCAGCAAAATGCGATAAGTAATGTGAATTGCAGAATTCAGTGAATCATCGAATCTTTGAACGCACATTGCGCCCGCCAGCATTCTGGCGGGCATGCCTGTTCGAGCGTCATTTCAACCCTCGACGAAGCC-GGTGTTGGGGCGCGGC---AGCTCGC--------CCGGCTCCGAAATTTAGTGGCGG-CCCGTTGCGGCGACCTCTGCGTAGTAA----TCGCACTGG-G-CAGCAGCGCGGCCACGCCGTAAAACCCCCGAC---------AAGGTTGACCTCGAATCAGGTAGGACTACCCGCTGAACTTAAGCATATCAAT--CGGAGG--

----

>AF108480.1 Simplicillium lamellicola isolate UAMH 4785 internal transcribed spacer 1, complete sequence

---CATTAAC-GAG-ATCAACTCCCACCCTTTGTGAAC--ACC--TTA--
CGTT-GCTTCGGCG--G-CGC------------------CAGGTCAACGCG-C-------AAACTC--CGAAA------------------
-----------TCTGAGTGG---
CAATGAATCAAAACTTTCAACAACGGATCTCTTGGTTCTGGCATCGATGAAGAACGCAGCAAAATGCGA
TAAGTAATGTGAATTGCAGAATTCAGTGAATCATCGAATCTTTGAACGCACATTGCGCCCGCCAGCATT
CTGGCGGGCATGCCTGTTCGAGCGTCATTTCAACCCTCGACGAAGCC-GGTGTTGGGGCGCGGC--------
--AGCTCGC--------CCGGCTCCGAAATTTAGTGGCGG-
CCCGTTGCGGCGACCTCTGCGTAGTAA----TCGCACTGG-G-
CAGCAGCGCGGCCACGCCGTAAAACCCCCGAC---------
AAGGTTGACCTCGAATCAGGTAGGACTACCCGCTGAACTTAAGCATATCAAT--CGGAGG-----------------
----

>MH979338.1 Simplicillium sp. RVO-2018a culture URM<BRA_:7918 small subunit ribosomal RNA gene, partial sequence; internal transcribed spacer 1, 5.8S ribosomal RNA gene, and internal transcribed spacer 2, complete sequence; and large subunit ribosomal RNA gene, partial sequence

--------------------------------GAACCTGCGGATCATTATC-GAGTATCAACTCCCACCCTATGTGAA---ACC--
TT----GTT-GCTTCGGCG--G-CGC--------------------------------------AAACTCCT--------------------------------
---CTGAGTGG---
CAATGAATCAAAACTTTCAACAACGGATCTCTTGGTTCTGGCATCGATGAAGAACGCAGCGAAATGCG
ATAAGTAATGTGAATTGCAGAATTCAGTGAATCATCGAATCTTTGAACGCACATTGCGCCCGCCAGCAT
TCTGGCGGGCATGCCTGTTCGAGCGTCATTTCAACCCTCGGG----CC-GGCGTTGGGGACCGGC----------
-----------------------------------ACTGCC---------------------GACG----CCGCCCCCGAAATTCAGTGGCGG-
CCCTTCGAGGCGACCTCTGCGTAGTAA----TCGCACTGG-
GAAATCGAAGCGGCCACGCCGTAAAACACCCAAA---------
CAGGTTGACCTCGAATCAGGTAGGACTACCCGCTGAACTTAAGC---

>MF066034.1 Simplicillium sp. AMG-2017a isolate CDA_734 internal transcribed spacer 1, partial sequence; 5.8S ribosomal RNA gene and internal transcribed spacer 2, complete sequence; and large subunit ribosomal RNA gene, partial sequence

--------------------------------GAACCTGCGGATCATTATC-GAGTATCAACTCCCACCCTATGTGAA---ACC--
TT----GTT-GCTTCGGCG--G-CGC--------------------------------------AAACTC-----------------------------------
--CTGAGTGG---
CAATGAATCAAAACTTTCAACAACGGATCTCTTGGTTCTGGCATCGATGAAGAACGCAGCGAAATGCG

ATAAGTAATGTGAATTGCAGAATTCAGTGAATCATCGAATCTTTGAACGCACATTGCGCCCGCCAGTAT
TCTGGCGGGCATGCCTGTTCGAGCGTCATTTCAACCCTCGAG----CC-GGCGTTGGGGACCGGC----------
-------------------------------------ACTGCC----------------------GACG----CCGCCCCCGAAATTTAGTGGCGG-
CCCTCTGAGGCGACCTCTGCGTAGTAA----TCGCACTGG-
AACATTAGAGTGGCCACGCCGTAAAACACCCAACT--------
CAGGTTGACCTCGAATCAGGTAGGACTACCCGCTGAACTTAAGC--

>MF066035.1 Simplicillium sp. AMG-2017a isolate CDA_735 internal transcribed spacer 1, partial sequence; 5.8S ribosomal RNA gene and internal transcribed spacer 2, complete sequence; and large subunit ribosomal RNA gene, partial sequence

---GCGGATCATTATC-GAGTATCAACTCCCACCCTATGTGAA---ACC--TT--
--GTT-GCTTCGGCG--G-CGC---AAACTC---------------------------------------
CTGAGTGG---
CAATGAATCAAAACTTTCAACAACGGATCTCTTGGTTCTGGCATCGATGAAGAACGCAGCGAAATGCG
ATAAGTAATGTGAATTGCAGAATTCAGTGAATCATCGAATCTTTGAACGCACATTGCGCCCGCCAGTAT
TCTGGCGGGCATGCCTGTTCGAGCGTCATTTCAACCCTCGAG----CC-GGCGTTGGGGACCGGC----------
-------------------------------------ACTGCC----------------------GACG----CCGCCCCCGAAATTTAGTGGCGG-
CCCTCTGAGGCGACCTCTGCGTAGTAA----TCGCACTGG-
AACATTAGAGTGGCCACGCCGTAAAACACCCAACT--------
CAGGTTGACCTCGAATCAGGTAGGACTACCCGCTGAACTTAAGCAT---

>JQ410324.1 Simplicillium chinense strain LC1345 internal transcribed spacer 1, partial sequence; 5.8S ribosomal RNA gene and internal transcribed spacer 2, complete sequence; and 28S ribosomal RNA gene, partial sequence

---ACC--TT----GTT-GCTTCGGCGC-G-
CGC---------------------------------------C-AAACTC-------A-------------------------------CTGAGTGG-T-
CAATGAATCAAAACTTTCAACAACGGATCTCTTGGTTCTGGCATCGATGAAGAACGCAGCGAAATGCG
ATAAGTAATGTGAATTGCAGAATTCAGTGAATCATCGAATCTTTGAACGCACATTGCGCCCGCCAGCAT
TCTGGCGGGCATGCCTGTTCGAGCGTCATTTCAACCCTCGAG----TC-GGCGTTGGGGGACGGC----------
-------------------------------------ACTCCC----------------------GACG----CCGCCCCCGAAATCTAGTGGCGG-
CCCTCCGAGGCGACCTCTGCGTAGTAA----TCGCACCGG-AAGCTCAGAGCGGCCACGCCGTAAAAC-
CCCAAC---------CAGGTTGACCTCGAATCAGGTAGGACTACCCGCTGAACTTAAGCATATCAA------------
----

>JQ410323.1 Simplicillium chinense strain LC1342 18S ribosomal RNA gene, partial sequence; internal transcribed spacer 1, 5.8S ribosomal RNA gene, and internal transcribed spacer 2, complete sequence; and 28S ribosomal RNA gene, partial sequence

-----GTAAAAGTCGTAACAAGGTTCCGTTGGTGAACCAGCGGATCATTATC-GAG-
ATCAACTCCCACCCTATGTGAA---ACC--TT----GTT-GCTTCGGCGC-G-CGC-------------------------------------
--C-AAACTC-------A-----------------------------CTGAGTGG-T-
CAATGAATCAAAACTTTCAACAACGGATCTCTTGGTTCTGGCATCGATGAAGAACGCAGCGAAATGCG
ATAAGTAATGTGAATTGCAGAATTCAGTGAATCATCGAATCTTTGAACGCACATTGCGCCCGCCAGCAT
TCTGGCGGGCATGCCTGTTCGAGCGTCATTTCAACCCTCGAG----TC-GGCGTTGGGGGACGGC----------
-----------------------------------ACTCCC---------------------GACG----CCGCCCCCGAAATCTAGTGGCGG-
CCCTCCGAGGCGACCTCTGCGTAGTAA----TCGCACCGG-AAGCTCAGAGCGGCCACGCCGTAAAAC-
CCCAAC---------CAGG-TGACCTCGAATCA---

>KP034998.1 Simplicillium chinense strain EXF-8701 18S ribosomal RNA gene, partial sequence; internal transcribed spacer 1 and 5.8S ribosomal RNA gene, complete sequence; and internal transcribed spacer 2, partial sequence

--------------GTAACAAGGTTCCGTTGGTGAACCAGCGGATCATTATC-GAG-
ATCAACTCCCACCCTATGTGAA---ACC--TT----GTT-GCTTCGGCGC-G-CGC-------------------------------------
--C-AAACTC-------A-----------------------------CTGAGTGG-T-
CAATGAATCAAAACTTTCAACAACGGATCTCTTGGTTCTGGCATCGATGAAGAACGCAGCGAAATGCG
ATAAGTAATGTGAATTGCAGAATTCAGTGAATCATCGAATCTTTGAACGCACATTGCGCCCGCCAGCAT
TCTGGCGGGCATGCCTGTTCGAGCGTCATTTCAACCCTCGAG----TC-GGCGTTGGGGGACGGC----------
-----------------------------------ACTCCC---------------------GACG----CCGCCCCCGAAATCTAGTGGCGG-
CCCTCCGAGGCGACCTCTGCGTAGTAA----TCGCACCGG-AAGCTCAGAGCGGCCACGCCGTAAAAC-
CCCAA--

