# Supplementary material for: An efficient Agrobacterium tumefaciens-mediated transformation method for Simplicillium subtropicum (Hypocreales: Cordycipitaceae)
Source: Genet Mol Biol. 2021 Oct 1;44(3):e20210073. doi: 10.1590/1678-4685-GMB-2021-0073 (PMC8489804; doi:10.1590/1678-4685-GMB-2021-0073)
Supplement: Table S1 - [file 1415-4757-GMB-44-3-e20210073-s2.pdf]

Genetics and Molecular Biology
https://doi.org/10.1590/1678-4685-GMB-2021-0073

Supplementary Material to "An efficient *Agrobacterium tumefaciens*-mediated transformation method for *Simplicillium subtropicum* (Hypocreales: Cordycipitaceae)"

Table S1 - Primer sequences used in this work.

Primer name	Sequence (5' – 3')
ITS4	TCCTCCGCTTATTGATATGC
ITS5	GCAAGTAAAAGTCGTAACAAGG
gpdA_CDSKat_F	CGCTTGAGCAGACATCACCATGGTGGGTGAGGATAGC
TtrpC_CDSKat_R	GATTTCAGTAACGTTAAGTGTCAGCTGTGCCCCAGTTTG
pPZP_HindIII_gpdA_Kat_F	AGAGGCCTAGGCGCGCCACTGTACAGTGACCGGTGACT
pPZP_HindIII_trpC_Kat_R	AAACGACGGCCAGTGCCAGAGTAAAGAAGAGGAGCATG
pPZP_EcoRV_SUR_F	ACGAATTCTTAATTAAGATTCGACGTGCCAACGCCACAG
pPZP_EcoRV_SUR_R	CCCGGGTACCGAGCTCGATTCGACGTGAGAGCATGCAATTC
SUR_F	TCGACGTGCCAACGCCACAG
SUR_R	TCGACGTGAGAGCATGCAATTC
