# Supplementary material for: An efficient Agrobacterium tumefaciens-mediated transformation method for Simplicillium subtropicum (Hypocreales: Cordycipitaceae)
Source: Genet Mol Biol. 2021 Oct 1;44(3):e20210073. doi: 10.1590/1678-4685-GMB-2021-0073 (PMC8489804; doi:10.1590/1678-4685-GMB-2021-0073)
Supplement: Figure S1 - [file 1415-4757-GMB-44-3-e20210073-s3.pdf]

Genetics and Molecular Biology
https://doi.org/10.1590/1678-4685-GMB-2021-0073

Supplementary Material to "An efficient *Agrobacterium tumefaciens*-mediated transformation method for *Simplicillium subtropicum* (Hypocreales: Cordycipitaceae)"

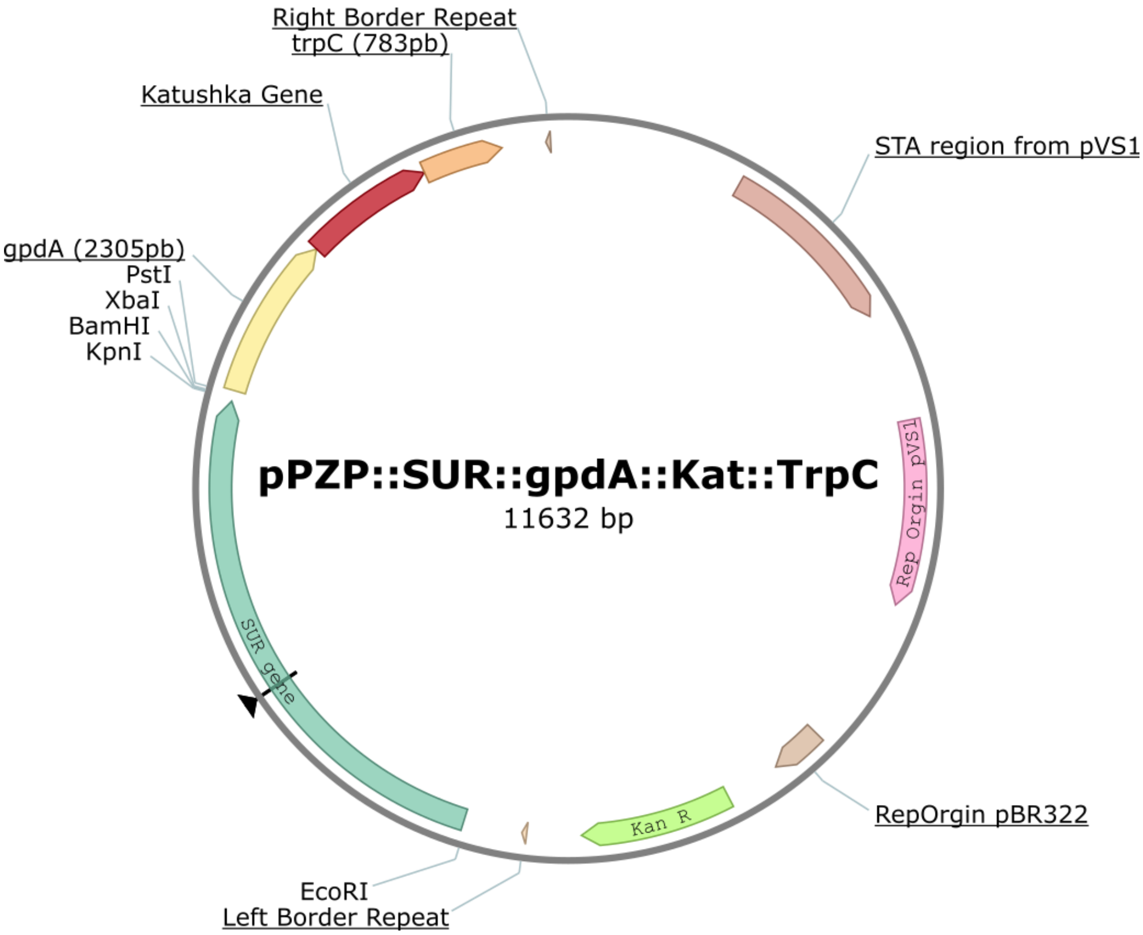

Figure S1 - pPZP201BK::SUR::gpdA::Kat::TrpC plasmid map.
