# Supplementary material for: An efficient Agrobacterium tumefaciens-mediated transformation method for Simplicillium subtropicum (Hypocreales: Cordycipitaceae)
Source: Genet Mol Biol. 2021 Oct 1;44(3):e20210073. doi: 10.1590/1678-4685-GMB-2021-0073 (PMC8489804; doi:10.1590/1678-4685-GMB-2021-0073)
Supplement: Supplementary Data S2 - [file 1415-4757-GMB-44-3-e20210073-s4.pdf]

Genetics and Molecular Biology
https://doi.org/10.1590/1678-4685-GMB-2021-0073

Supplementary Material to "An efficient *Agrobacterium tumefaciens*-mediated transformation method for *Simplicillium subtropicum* (Hypocreales: Cordycipitaceae)"

Supplementary Data S2 - pPZP201BK::SUR::gpdA::Kat::TrpC plasmid sequence.

Supplementary Data S2

Plasmid sequence:

agtactttgatccaacccctccgctgctatagtgcagtcggcttctgacgttcagtgcagccgtcttctgaaaacgacatgtcgca

caagtcctaagttacgcgacaggctgccgccctgcccttttcctggcgttttcttgtcgcgtgttttagtcgcataaagtagaatac

ttgcgactagaaccggagacattacgccatgaacaagagcgccgccgctggcctgctgggctatgcccgcgtcagcaccga

cgaccaggacttgaccaaccaacgggccgaactgcacgcggccggctgcaccaagctgttttccgagaagatcaccggca

ccaggcgcgaccgcccggagctggccaggatgcttgaccacctacgccctggcgacgttgtgacagtgaccaggctagac

cgcctggcccgcagcacccgcgacctactggacattgccgagcgcatccaggaggccggcgcgggcctgcgtagcctgg

cagagccgtgggccgacaccaccacgccggccggccgcatggtgttgaccgtgttcgccggcattgccgagttcgagcgtt

ccctaatcatcgaccgcacccggagcgggcgcgaggccgccaaggcccgaggcgtgaagtttggcccccgccctaccctc

accccggcacagatcgcgcacgcccgcgagctgatcgaccaggaaggccgcaccgtgaaagaggcggctgcactgcttg

gcgtgcatcgctcgaccctgtaccgcgcacttgagcgcagcgaggaagtgacgcccaccgaggccaggcggcgcggtgc

cttccgtgaggacgcattgaccgaggccgacgccctggcggccgccgagaatgaacgccaagaggaacaagcatgaaac

cgcaccaggacggccaggacgaaccgtttttcattaccgaagagatcgaggcggagatgatcgcggccgggtacgtgttcg

agccgcccgcgcacgtctcaaccgtgcggctgcatgaaatcctggccggtttgtctgatgccaagctggcggcctggccggc

cagcttggccgctgaagaaaccgagcgccgccgtctaaaaaggtgatgtgtatttgagtaaaacagcttgcgtcatgcggtcg

ctgcgtatatgatgcgatgagtaaataaacaaatacgcaaggggaacgcatgaaggttatcgctgtacttaaccagaaaggcg

ggtcaggcaagacgaccatcgcaacccatctagcccgcgccctgcaactcgccggggccgatgttctgttagtcgattccgat

ccccagggcagtgcccgcgattgggcggccgtgcgggaagatcaaccgctaaccgttgtcggcatcgaccgcccgacgat

tgaccgcgacgtgaaggccatcggccggcgcgacttcgtagtgatcgacggagcgccccaggcggcggacttggctgtgt

ccgcgatcaaggcagccgacttcgtgctgattccggtgcagccaagcccttacgacatatgggccaccgccgacctggtgga

gctggttaagcagcgcattgaggtcacggatggaaggctacaagcggcctttgtcgtgtcgcgggcgatcaaaggcacgcg

catcggcggtgaggttgccgaggcgctggccgggtacgagctgcccattcttgagtcccgtatcacgcagcgcgtgagctac

ccaggcactgccgccgccggcacaaccgttcttgaatcagaacccgagggcgacgctgcccgcgaggtccaggcgctgg

ccgctgaaattaaatcaaaactcatttgagttaatgaggtaaagagaaaatgagcaaaagcacaaacacgctaagtgccggcc

gtccgagcgcacgcagcagcaaggctgcaacgttggccagcctggcagacacgccagccatgaagcgggtcaactttcag

ttgccggcggaggatcacaccaagctgaagatgtacgcggtacgccaaggcaagaccattaccgagctgctatctgaataca

tcgcgcagctaccagagtaaatgagcaaatgaataaatgagtagatgaattttagcggctaaaggaggcggcatggaaaatc

aagaacaaccaggcaccgacgccgtggaatgccccatgtgtggaggaacgggcggttggccaggcgtaagcggctgggtt

gtctgccggccctgcaatggcactggaacccccaagcccgaggaatcggcgtgacggtcgcaaaccatccggcccggtac

aaatcggcgcggcgctgggtgatgacctggtggagaagttgaaggccgcgcaggccgcccagcggcaacgcatcgaggc

agaagcacgccccggtgaatcgtggcaagcggccgctgatcgaatccgcaaagaatcccggcaaccgccggcagccggt

gcgccgtcgattaggaagccgcccaagggcgacgagcaaccagatttttttcgttccgatgctctatgacgtgggcacccgcg

atagtcgcagcatcatggacgtggccgttttccgtctgtcgaagcgtgaccgacgagctggcgaggtgatccgctacgagctt

ccagacgggcacgtagaggtttccgcagggccggccggcatggccagtgtgtgggattacgacctggtactgatggcggttt

cccatctaaccgaatccatgaaccgataccgggaagggaagggagacaagcccggccgcgtgttccgtccacacgttgcg

gacgtactcaagttctgccggcgagccgatggcggaaagcagaaagacgacctggtagaaacctgcattcggttaaacacc

acgcacgttgccatgcagcgtacgaagaaggccaagaacggccgcctggtgacggtatccgagggtgaagccttgattagc

cgctacaagatcgtaaagagcgaaaccgggcggccggagtacatcgagatcgagctagctgattggatgtaccgcgagatc

acagaaggcaagaacccggacgtgctgacggttcaccccgattactttttgatcgatcccggcatcggccgttttctctaccgc

ctggcacgccgcgccgcaggcaaggcagaagccagatggttgttcaagacgatctacgaacgcagtggcagcgccggag

agttcaagaagttctgtttcaccgtgcgcaagctgatcgggtcaaatgacctgccggagtacgatttgaaggaggaggcggg

gcaggctggcccgatcctagtcatgcgctaccgcaacctgatcgagggcgaagcatccgccggttcctaatgtacggagca

gatgctagggcaaattgccctagcaggggaaaaaggtcgaaaaggtctctttcctgtggatagcacgtacattgggaacccaa

agccgtacattgggaaccggaacccgtacattgggaacccaaagccgtacattgggaaccggtcacacatgtaagtgactga

tataaaagagaaaaaaggcgatttttccgcctaaaactctttaaaacttattaaaactcttaaaacccgcctggcctgtgcataact

gtctggccagcgcacagccgaagagctgcaaaaagcgcctacccttcggtcgctgcgctccctacgccccgccgcttcgcg

tcggcctatcgcggccgctggccgctcaaaaatggctggcctacggccaggcaatctaccagggcgcggacaagccgcgc

cgtcgccactcgaccgccggcgcccacatcaaggcaccctgcctcgcgcgtttcggtgatgacggtgaaaacctctgacaca

tgcagctcccggagacggtcacagcttgtctgtaagcggatgccgggagcagacaagcccgtcagggcgcgtcagcgggt

gttggcgggtgtcggggcgcagccatgacccagtcacgtagcgatagcggagtgtatactggcttaactatgcggcatcaga

gcagattgtactgagagtgcaccatatgcggtgtgaaataccgcacagatgcgtaaggagaaaataccgcatcaggcgctctt

ccgcttcctcgctcactgactcgctgcgctcggtcgttcggctgcggcgagcggtatcagctcactcaaaggcggtaatacgg

ttatccacagaatcaggggataacgcaggaaagaacatgtgagcaaaaggccagcaaaaggccaggaaccgtaaaaaggc

cgcgttgctggcgtttttccataggctccgcccccctgacgagcatcacaaaaatcgacgctcaagtcagaggtggcgaaacc

cgacaggactataaagataccaggcgtttccccctggaagctccctcgtgcgctctcctgttccgaccctgccgcttaccggat

acctgtccgcctttctcccttcgggaagcgtggcgctttctcatagctcacgctgtaggtatctcagttcggtgtaggtcgttcgct

ccaagctgggctgtgtgcacgaaccccccgttcagcccgaccgctgcgccttatccggtaactatcgtcttgagtccaacccg

gtaagacacgacttatcgccactggcagcagccactggtaacaggattagcagagcgaggtatgtaggcggtgctacagagt

tcttgaagtggtggcctaactacggctacactagaaggacagtatttggtatctgcgctctgctgaagccagttaccttcggaaa

aagagttggtagctcttgatccggcaaacaaaccaccgctggtagcggtggtttttttgtttgcaagcagcagattacgcgcaga

aaaaaaggatctcaagaagatcctttgatcttttctacggggtctgacgctcagtggaacgaaaactcacgttaagggattttggt

catgcaggatcatgaattaattcttagaaaaactcatcgagcatcaaatgaaactgcaatttattcatatcaggattatcaataccat

atttttgaaaaagccgtttctgtaatgaaggagaaaactcaccgaggcagttccataggatggcaagatcctggtatcggtctgc

gattccgactcgtccaacatcaatacaacctattaatttcccctcgtcaaaaataaggttatcaagtgagaaatcaccatgagtga

cgactgaatccggtgagaatggcaaaagtttatgcatttctttccagacttgttcaacaggccagccattacgctcgtcatcaaaa

tcactcgcatcaaccaaaccgttattcattcgtgattgcgcctgagcgagacgaaatacgcgatcgctgttaaaaggacaattac

aaacaggaatcgaatgcaaccggcgcaggaacactgccagcgcatcaacaatattttcacctgaatcaggatattcttctaata

cctggaatgctgttttcccggggatcgcagtggtgagtaaccatgcatcatcaggagtacggataaaatgcttgatggtcggaa

gaggcataaattccgtcagccagtttagtctgaccatctcatctgtaacatcattggcaacgctacctttgccatgtttcagaaaca

actctggcgcatcgggcttcccatacaatcgatagattgtcgcacctgattgcccgacattatcgcgagcccatttatacccatat

aaatcagcatccatgttggaatttaatcgcggcctagagcaagacgtttcccgttgaatatggctcataacaccccttgtattactg

tttatgtaagcagacagttttattgttcatgatctggatcacaggcagcaacgctctgtcatcgttacaatcaacatgctaccctcc

gcgagatcatccgtgtttcaaacccggcagcttagttgccgttcttccgaatagcatcggtaacatgagcaaagtctgccgcctt

acaacggctctcccgctgacgccgtcccggactgatgggctgcctgtatcgagtggtgattttgtgccgagctgccggtcggg

gagctgttggctggctggtggcaggatatattgtggtgtaaacaaattgacgcttagacaacttaataacacattgcggacgtttt

taatgtactgaattaacgccgaattgctctagccaatacgcaaaccgcctctccccgcgcgttggccgattcattaatgcagctg

gcacgacaggtttcccgactggaaagcgggcagtgagcgcaacgcaattaatgtgagttagctcactcattaggcaccccag

gctttacactttatgcttccggctcgtatgttgtgtggaattgtgagcggataacaatttcacacaggaaacagctatgacatgatt

acgaattcttaattaagattcgacgtgccaacgccacagtgccccacatctcccggctggattcaccgttgagcggggattgtgt

ggagggacttggtcgaaaatttgtgaacctccgatggcggcaaacatgactccacgattcacagcccaaaggcctggccctg

cttcaattgtcatcgtctgacaggtccaggaaaaatagttagctggaggctcgttactttttttcccgcttcaacggccaagcaag

caagctcatcgtctctcgttcttttctccattattgacgactgagaacagattcgaaatgcttcgtactgttggccgcaaagccctg

aggggctcatccaagggatgttcacgaaccatctcgactctcaagcccgccacggcaactattgccaagcccggcagcagg

accctttcgacgccagcgacggcaacagcaacgtaagtcagaatcacgagcgagcgaacaaacaggcagagttacaaacg

ccatcactacgccttgtcttttcgtctctgttacgtgtcgccacccccatgcgagctacacgtctgcgcctgaagtcacaaggctt

gcaatttcatgacgatgaacaacactgacacttctgttacagagcacctcgaactaagcccagcgccagcttcaatgctcgccg

cgatccccagcctttggtcaaccctcgctcaggtgaggcagacgaatcgtaagttgcgccaacaccgttcctacaccgccaag

acccataccgccgttgcatttggacaagcacaagactgatttgaccgtggcatagattcattggcaagacgggaggagagattt

tccacgagatgatgctgaggcaaaacgtcaagcacatttgtaagcgtctaattctacaaattcctaccctcgattcaaccacatat

cttgacacatgagtcacagtcggttaccctggcggtgctatccttcccgtgttcgacgcgatctacaactcgaagcacatcgact

ttgttctgcccaagcatgagcaaggcgccggccacatggcagagggctatgctcgcgcttcaggcaaacccggcgttgttctc

gtcacctccggccccggtgccacaaatgtcatcactcccatggccgacgctcttgccgacggtacacctctggttgtattctca

ggacaggttgttacctctgatattggaagcgacgccttccaggaggccgacgtcataggcatctcccggtcttgcaccaagtg

gaacgtcatggttaagagcgctgacgagctcccgaggagaattaacgaggcctttgagattgccaccagtgggcgacctgg

gcctgtcttggtcgatcctgccaaggacgtcacggctagtgtgctgaggagggctatccccaccgagacctcgattccttctatt

agcgcagcagcacgggctgtccaagaggcaggccgaaagcagcttgagcactccatcaaacgcgtagccgatctcgtcaa

cattgccaagaagcccgtcatatatgccggccaaggtgtcattttgtcggaaggcggcgttgaacttctcaaggcgcttgccga

caaggcctcgattcctgtcaccaccactctgcatggtctgggagcctttgacgagctcgacgagaaggcactgcacatgcttg

gtatgcacggttcggcttatgccaacatgtccatgcaagaggccgatttgatcattgcccttggtggccgcttcgatgaccgtgt

cactggcagcatccccaaatttgctcctgccgccaagctagctgctgctgaaggacgcggaggtattgtccacttcgagattat

gcccaagaacatcaacaaggtcgtccaagcaacagaggccattgagggcgacgttgcttcgaacttgaagctgttgctcccc

aagattgaacaacgatccatgaccgatcgcaaggagtggttcgaccagatcaaggagtggaaggagaagtggcctctgtca

cattatgagagggccgagcgtagtggtctcatcaagcctcagactctgatcgaggagctgagcaacctgactgctgaccgca

aggacatgacctacatcacaaccggtgttggccagcaccaaatgtggacagcacaacatttcaggtggaggcacccacggtc

catgatcacctctggcggtttgggaaccatgggatatggtctgccggcagcgattggcgccaaggttgctaggccagatgcttt

ggtcattgacatcgacggcgacgcatcgttcaacatgactctgacagagctttcgacggcggcacagttcaacattggcgtca

aggtcattgtcttgaacaacgaggagcagggaatggtgacccaatggcagaacttgttctacgaggaccgctactcacataca

caccagcgcaacccagacttcatgaagctcgccgatgcaatggacgttcaacatcgccgtgtttcgaagcctgacgatgtcgg

tgatgctctgacgtggctgatcaacaccgacggccccgctctgcttgaggtgatgactgataagaaggttcctgttctgcccatg

gtgcccggaggtaacggcctgcacgagttcatcacgtttgatgccagtaagtaatatatccaatgcactcctgtggcccattttg

agccttgggtatgattctagttcaacttgctaaccagtgcatcaactcgtataggcaaggataagcaacggagagagctgatgc

gcgcgaggacgaatggcctgcacggttaagttgataatgggaattgattattgcacgggaattgcatgctctcacgtcgaatcg

agctcggtacccggggatcctctagagtcgacctgcagaggcctaggcgcgccactgtacagtgaccggtgactctttctgg

catgcggagagacggacggacgcagagagaagggctgagtaataagccactggccagacagctctggcggctctgaggt

gcagtggatgattattaatccgggaccggccgcccctccgccccgaagtggaaaggctggtgtgcccctcgttgaccaagaa

tctattgcatcatcggagaatatggagcttcatcgaatcaccggcagtaagcgaaggagaatgtgaagccaggggtgtatagc

cgtcggcgaaatagcatgccattaacctaggtacagaagtccaattgcttccgatctggtaaaagattcacgagatagtaccttc

tccgaagtaggtagagcgagtacccggcgcgtaagctccctaattggcccatccggcatctgtagggcgtccaaatatcgtgc
ctctcctgctttgcccggtgtatgaaaccggaaaggccgctcaggagctggccagcggcgcagaccgggaacacaagctg
gcagtcgacccatccggtgctctgcactcgacctgctgaggtccctcagtccctggtaggcagctttgccccgtctgtccgccc
ggtgtgtcggcggggttgacaaggtcgttgcgtcagtccaacatttgttgccatattttcctgctctccccaccagctgctcttttct
tttctctttcttttcccatcttcagtatattcatcttcccatccaagaacctttatttcccctaagtaagtactttgctacatccatactcca
tccttcccatcccttattcctttgaacctttcagttcgagctttcccacttcatcgcagcttgactaacagctaccccgcttgagcag
acatcaccTCAGCTGTGCCCCAGTTTGCTAGGCAGGTCGCAGTACCTGGCCACAG
CCATCTCGTGCTGCTCGACGTAGGTCTCTTTGTCGGCCTCCTTGATTCTTTCC
AGTCTCCTGTCCACGAAGTAGAAGCCGGGCATCTTGAGGTTCTTAGCGGGTT
TCTTGGATCTGTATGTGGTCTTGAGGGAGCAGTGCAGGTAGCCCCCGCCCAC
GAGCTTCAGGGCCATCTGGCTATGGCCTCTCAGGCCGCTGTCAGCGGGGTA
CAGCATCTCGGTGCTGGCCTCCCAGCCGAGTGTTTTCTTCTGCATCACAGGG
CCGTTGGATGGGAAGTTCACCCCGTTGATCTTGACGTTGTAGATGAGGCAGC
CGTTCTGGAGGCTGGTGTCCTGGGTAGCGGTCAGCACGCCCCCGTCTTCGTA
TGTGGTGATCCTCTCCCATGTGAAGCCCTCAGGGAAGGACTGCTTAAAGAA
GTCGGGGATGCCCTGGGTGTGGTTGATAAAGGTTTTGCTGCCGTACATGAA
GCTGGTAGCCAGGATGTCGAAGGCGAAGGGGAGAGGGCCGCCCTCGACCA
CCTTGATCTTCATGGTCTGGGTGCCCTCGTAGGGCTTGCCTTCGCCCTCGGA
TGTGCACTTGAAGTGGTGGTCGTTCACGGTGCCCTCCATGTACAGTTTCATG
TGCATGTTCTCGGTGATCAGCACGCTATCCTCACCCACCATcacttaacgttactgaaat
catcaaacagcttgacgaatctggatataagatcgttggtgtcgatgtcagctccggagttgagacaaatggtgttcaggatctc
gataagatacgttcatttgtccaagcagcaaagagtgccttctagtgatttaatagctccatgtcaacaagaataaaacgcgttttc
gggtttacctcttccagatacagctcatctgcaatgcattaatgcattgactgcaacctagtaacgccttncaggctccggcgaa
gagaagaatagcttagcagagctattttcattttcgggagacgagatcaagcagatcaacggtcgtcaagagacctacgagac
tgaggaatccgctcttggctccacgcgactatatatttgtctctaattgtactttgacatgctcctcttctttactctggcactggccgt

cgttttacaacgtcgtgactgggaaaaccctggcgttacccaacttaatcgccttgcagcacatccccctttcgccagctggcgt

aatagcgaagaggcccgcaccgatcgcccttcccaacagttgcgcagcctgaatggcgaatgagcttgagcttggatcagat

tgtcgtttcccgccttcagtttaaactatcagtgtttgacaggatatattggcgggtaaacctaagagaaaagagcgtttattagaa

taacggatatttaaaagggcgtgaaaaggtttatccgttcgtccatttgtatgtgcatgccaaccacagggttcccctcgggatca

a
